# Inhibition of USP9X Downregulates JAK2-V617F and Induces Apoptosis Synergistically with BH3 Mimetics Preferentially in Ruxolitinib-Persistent JAK2-V617F-Positive Leukemic Cells

**DOI:** 10.3390/cancers12020406

**Published:** 2020-02-10

**Authors:** Hiroki Akiyama, Yoshihiro Umezawa, Daisuke Watanabe, Keigo Okada, Shinya Ishida, Ayako Nogami, Osamu Miura

**Affiliations:** 1 Department of Hematology, Graduate School of Medical and Dental Sciences, Tokyo Medical and Dental University, Tokyo 113-8519, Japan; hakihema@tmd.ac.jp (H.A.); umehema@tmd.ac.jp (Y.U.); dwathema@tmd.ac.jp (D.W.); keighema@tmd.ac.jp (K.O.); s-ishida.hema@yokohama.jrc.or.jp (S.I.); nogahema@tmd.ac.jp (A.N.); 2 Department of Clinical Laboratory, Medical Hospital, Tokyo Medical and Dental University, 1-5-45 Yushima, Bunkyoku, Tokyo 113-8519, Japan

**Keywords:** JAK2-V617F, USP9X, WP1130, ruxolitinib, myeloproliferative neoplasms, BH3 mimetics, oxidative stress

## Abstract

JAK2-V617F plays a key role in the pathogenesis of myeloproliferative neoplasm. However, its inhibitor ruxolitinib has shown limited clinical efficacies because of the ruxolitinib-persistent proliferation of JAK2-V617F-positive cells. We here demonstrate that the USP9X inhibitor WP1130 or EOAI3402143 (G9) inhibited proliferation and induced apoptosis more efficiently in cells dependent on JAK2-V617F than on cytokine-activated JAK2. WP1130 preferentially downregulated activated and autophosphorylated JAK2-V617F by enhancing its K63-linked polyubiquitination and inducing its aggresomal translocation to block downstream signaling. Furthermore, JAK2-V617F associated physically with USP9X in leukemic HEL cells. Induction of apoptosis by inhibition of USP9X was mediated through the intrinsic mitochondria-mediated pathway, synergistically enhanced by BH3 mimetics, prevented by overexpression of Bcl-xL, and required oxidative stress to activate stress-related MAP kinases p38 and JNK as well as DNA damage responses in HEL cells. Although autophosphorylated JAK2-V617F was resistant to WP1130 in the ruxolitinib-persistent HEL-R cells, these cells expressed Bcl-2 and Bcl-xL at lower levels and showed an increased sensitivity to WP1130 as well as BH3 mimetics as compared with ruxolitinib-naive HEL cells. Thus, USP9X represents a promising target along with anti-apoptotic Bcl-2 family members for novel therapeutic strategies against JAK2-V617F-positive myeloproliferative neoplasms, particularly under the ruxolitinib persistence conditions.

## 1. Introduction

The Janus kinases (JAKs) are nonreceptor tyrosine kinases involved in transduction of cytokine-mediated signaling in various hematopoietic cells [1]. Among the four members (JAK1, 2, 3, and TYK2), JAK2 is coupled with receptors for erythropoietin (Epo), thrombopoietin (Tpo), and granulocyte-colony stimulating factor (G-CSF) and plays a critical role in hematopoiesis. JAK2 activated by these cytokine receptors regulates proliferation, differentiation, and survival of myeloid cells through stimulation of various signaling events, such as activation of STATs and the Ras/MEK/Erk and PI3K/Akt pathways. Thus, aberrant activation of these pathways results in cytokine-independent cell growth seen in hematopoietic malignancies including myeloproliferative neoplasms (MPNs) [2,3]. The somatic mutation JAK2-V617F is the most frequently observed driver mutation in BCR/ABL-negative MPNs, such as polycythemia vera (PV) and primary myelofibrosis (PMF) [4]. Some cases of PMF or PV progress and transform into secondary acute myeloid leukemia (post-MPN sAML) with its frequency increased up to 20% in patients treated with chemotherapy. Several JAK2-V617F-positive cell lines have been derived from patients with post-MPN sAML, including PVTL-1 and PVTL-2 as we established previously, to be used in various studies on JAK2-V617F-mediated signaling and leukemogenesis [5,6,7]. The other predominant driver mutations seen in MPNs involving the Tpo receptor c-MPL or calreticulin (CALR) constitutively stimulate JAK2 and its downstream pathways through constitutive activation of c-MPL, underscoring the importance of aberrant JAK2 signaling in the pathogenesis of MPNs [3]. 

A number of JAK2 inhibitors have been under clinical trials for MPNs, and only the JAK1/JAK2 inhibitor ruxolitinib has been in clinical use in general practice for PMF or PV mainly to reduce splenomegaly and various symptoms and possibly to improve survival of these patients [8]. However, JAK inhibition by ruxolitinib has not been associated with significant decrease in JAK2-V617F mutant clone in most MPNs patients. Furthermore, ruxolitinib has shown very limited efficacies against post-MPN sAML, which bears the uniformly dismal prognosis with median survival of less than 6 months [9,10]. It is presumed that its failure to reduce disease burden is not due to acquired drug resistance mutations but rather due to ruxolitinib-persistent proliferation and survival of JAK2-V617F-positive MPN cells under the condition of chronic JAK2 inhibition as well as its inhibitory effect on cytokine-activated JAK2 in normal hematopoietic cells. In this regard, it has been demonstrated that JAK2-V617F-positive post-MPN sAML cell lines readily gain the ability to proliferate persistently in the presence of ruxolitinib after a long-term exposure to gradually increasing concentrations of the inhibitor [11,12,13]. Thus, development of newer therapeutic strategies is urgently needed for MPNs and, particularly, post-MPN sAML. 

The ubiquitin–proteasome system (UPS) regulates various physiological processes via post-translational modification of cellular proteins [14]. Since cancer cells also depend on functional UPS, modulation of target proteins through interfering UPS can be a promising therapeutic approach [15]. Different types of ubiquitination have distinct effects on the target proteins. For example, the most prevalent polyubiquitination through K48-linkage targets proteins for proteasomal degradation, while that through K63-linkage is mainly associated with protein–protein interaction and intracellular localization. WP1130 is a partially selective deubiquitinase (DUB) inhibitor that inhibits the deubiquitinating activities of USP9X, USP5, USP14, and UCHL5 (UCH37) [16]. WP1130 was first reported to block the aberrant BCR-ABL tyrosine kinase expressed in chronic myelogenous leukemia (CML) cells by increasing K63-linked polyubiquitination to induce its translocation to the aggresome [17,18], which is a perinuclear inclusion body containing aggregates of misfolded, ubiquitinated proteins, and chaperones [19]. Thus, WP1130 induced apoptosis in CML cells. It has been also reported that WP1130 blocks USP9X to promote apoptosis by sensitizing various solid tumor cells to ABT-737 and navitoclax (ABT-263), BH3 mimetics that inhibit anti-apoptotic Bcl-xL, Bcl-2, and Bcl-w [20,21]. In addition, we have recently reported that inhibition of USP9X by WP1130 as well as EOAI3402143 (G9) induced aggresomal translocation of the mutant receptor kinase FLT3 internal tandem duplication (FLT3-ITD) expressed in AML cells to block the downstream signaling events and induced oxidative stress, thus cooperatively activating the intrinsic mitochondria-mediated apoptotic pathway, which was synergistically enhanced by BH3 mimetics and prevented by overexpression of Bcl-xL or Mcl-1 [22]. On the other hand, there has been a report that investigated the effect of WP1130 on JAK2, in which Kapuria, et al. demonstrated that WP1130 downregulated cytokine-activated JAK2 as well as JAK2-V617F and downstream STAT5 signaling pathways [23]. However, the details of the mechanisms involved in WP1130-induced cytotoxic effects and the difference between its effects on wild-type JAK2 and on JAK2-V617F remain to be clarified.

In this study, we examine the efficacy of the USP9X inhibitors WP1130 and G9 on cells expressing wild-type JAK2 or JAK2-V617F, including the ruxolitinib-persistent cells, comparably, to explore the therapeutic potentials of USP9X inhibition against JAK2-V617F-driven myeloid neoplasms. We also investigate the molecular mechanisms involved in WP1130-induced apoptosis with respect to the involvement of oxidative stress and anti-apoptotic Bcl-2 family proteins to seek for strategies to enhance the therapeutic efficacy.

## 2. Results

### 2.1. The USP9X Inhibitor WP1130 or G9 Induces Apoptosis More Prominently in JAK2-V617F-Dependent Cells than in Cells Dependent on BCR/ABL or Cytokine-Activated JAK2 

**Figure 1:**
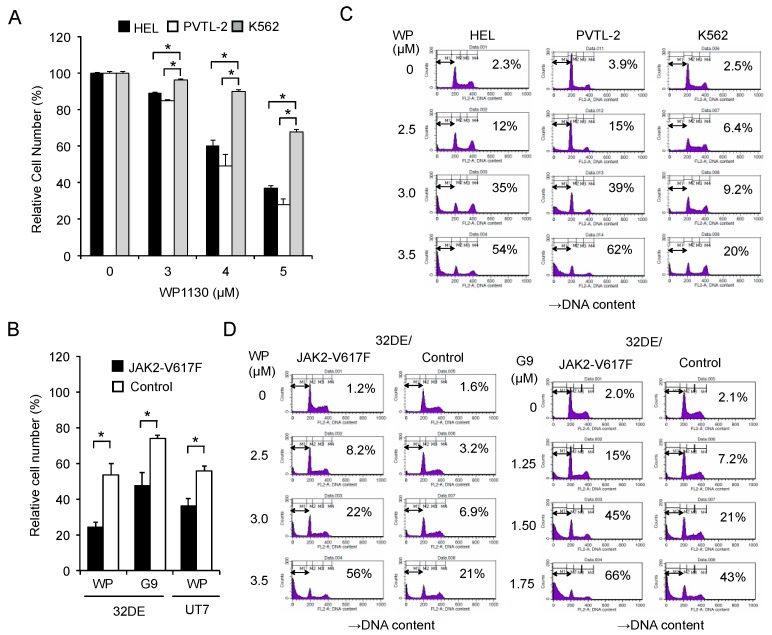
The USP9X inhibitor WP1130 or G9 induces apoptosis more prominently in JAK2-V617F-dependent cells than in cells dependent on BCR/ABL or cytokine-activated JAK2. (**A**) HEL, PVTL-2, and K562 cells were treated with indicated concentrations of WP1130 for 24 h, and viable cell numbers were measured by the CCK-8 colorimetric assay. Each data point represents the mean of triplicate cultures, with error bars indicating standard errors, and is expressed as percentage of the cell numbers cultured without WP1130. The asterisks indicate significant differences between K562 and HEL or PVTL-2 determined by one-way ANOVA followed by Dunnett’s post-hoc test (* *p* < 0.05). (**B**) 32DE or UT7 cells transduced with either JAK2-V617F or empty vector (Control) and cultured with Epo were left untreated as control or treated for 24 h with 3 μM WP1130 (WP) or 2 µM G9, as indicated, in triplicate, and viable cell numbers were measured. The asterisks indicate significant differences between JAK2-V617F and control cells determined by Student’s t-test (* *p* < 0.05). (**C**) HEL, PVTL-2, and K562 cells were treated for 24 h with indicated concentrations of WP1130. Cells were analyzed for cellular DNA content by flow cytometry. Percentages of apoptotic cells with the sub-G1 DNA content are indicated. (**D**) 32DE/JAK2-V617F cells (JAK2-V617F) or vector-control cells (Control) cultured with Epo were treated for 5 h with indicated concentrations of WP1130 or G9 and analyzed.

First, we examined the anti-proliferative effect of WP1130 against JAK2-V617F-driven leukemic cell lines. WP1130 inhibited proliferation of HEL and PVTL-2 post-MPN sAML cell lines harboring endogenous JAK2-V617F in dose-dependent manners (Figure 1A). The antiproliferative effects against these cell lines were more prominent than those against BCR-ABL-driven K562, a leukemic cell line derived from patients with CML in blastic phase reported to be susceptible to WP1130 [17,18]. 

To evaluate the impact of JAK2-V617F mutant on the antiproliferative effect of WP1130, we then utilized murine 32DE cells transduced with either exogenous JAK2-V617F or a control vector. Proliferation of cytokine-independent 32DE/JAK2-V617F cells was more significantly suppressed by WP1130 or G9 than that of control cells (Figure 1B), whose proliferation as well as survival is dependent on Epo-activated wild-type JAK2 [24,25]. Similarly, these inhibitors more significantly inhibited JAK2-V617F-dependent proliferation of the human leukemic UT7 cells transduced with this mutant than Epo-dependent proliferation of the vector-control cells (Figure 1B and Figure S1).

Next, we examined the antileukemic effect of WP1130 from the viewpoint of apoptosis induction. WP1130 induced apoptosis in HEL and PVTL-2 cells in dose-dependent manners and more prominently than in K562 cells, as judged by increases in the population of cells with sub-G1 cellular DNA content (Figure 1C). Furthermore, WP1130, as well as G9, induced apoptosis more prominently in 32DE/JAK2-V617F cells than in the vector control cells (Figure 1D). Together, these results suggest that inhibition of USP9X affects cells transformed by JAK2-V617F more prominently than those dependent on cytokine-activated wild-type JAK2 or BCR/ABL, mainly by inducing apoptosis.

### 2.2. WP1130 Enhances K63-Linked Polyubiquitination and Preferentially Downregulates the Phosphorylated Form of JAK2-V617F to Inhibit Downstream Signaling

**Figure 2:**
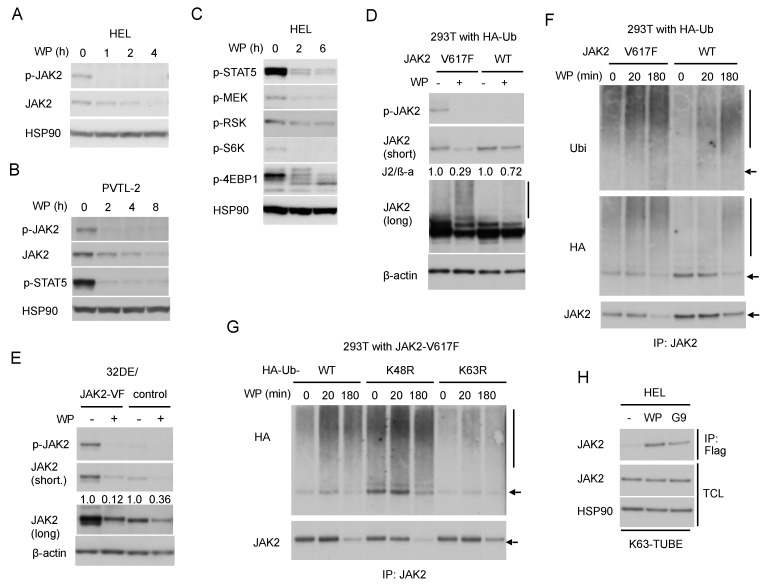
WP1130 enhances K63-linked polyubiquitination and preferentially downregulates the phosphorylated form of JAK2-V617F to inhibit downstream signaling. (**A**,**B**,**C**) HEL (**A**,**C**) or PVTL-2 (**B**) were treated with 5 µM WP1130 (WP) for indicated times. Cells were lysed and subjected to immunoblot analysis with antibodies against indicated proteins. HSP90 was used for a loading control. (**D**) 293T cells transfected with plasmids coding for either JAK2-V617F (V617F) or wild-type JAK2 (WT) and HA-tagged ubiquitin were treated for 3 h with or without 5 µM WP1130, as indicated, and analyzed. β-actin was used for a loading control. Short and long exposure results are shown where indicated. Relative expression levels of JAK2 as compared with those in untreated cells analyzed by densitometry and normalized by that of ß-actin (J2/β -a) are indicated. The vertical line indicates the smeary pattern characteristic of polyubiquitination. (**E**) 32DE/JAK2-V617F cells or vector control cells cultured with Epo were treated with or without 5 µM WP1130 for 2 h, as indicated, and analyzed. (**F**) 293T cells transduced with HA-tagged ubiquitin along with either JAK2-V617F or wild-type JAK2 were treated for indicated times with 5 µM WP1130 and lysed. Immunoprecipitates (IP) obtained with anti-JAK2 were analyzed. The arrow indicates the position corresponding to JAK2. (**G**) 293T cells transduced with JAK2-V617F along with HA-tagged ubiquitin (WT) or its K48R or K63R mutant were treated with 5 µM WP1130 for indicated times and analyzed. (**H**) HEL cells were left untreated as control or treated for 30 min with 5 µM WP1130 or 3 µM G9 as indicated. K63-polyubiquitinated proteins were isolated by immunoprecipitation with anti-Flag after incubation of cell lysates with FLAG-K63-TUBE (K63-TUBE). Immunoprecipitates, as well as total cell lysates (TCL), were analyzed.

Next, we examined the effect of WP1130 against JAK2 and its downstream signaling. In line with the previous report by Kapuria et al. [23], WP1130 downregulated the JAK2-V617F protein level time-dependently in HEL and PVTL-2 cells (Figure 2A,B), which are both homozygous for the JAK2-V617F mutation [5,6]. Importantly, activated JAK2-V617F phosphorylated at Y1007/1008 was much more rapidly downregulated compared with total JAK2-V617F in these cells and accompanied by inhibition of downstream activation of STAT5 as well as the MEK/RSK and mTORC1/S6K/4EBP1 pathways (Figure 2B,C). 

To compare the effect of WP1130 on JAK2-V617F and wild-type JAK2, we then examined 293T cells transiently transduced with either of them along with ubiquitin. The WP1130-induced downregulation was more prominently observed for JAK2-V617F as compared with wild-type JAK2 (Figure 2D). Similarly, JAK2 was downregulated more rapidly in 32DE cells expressing exogenous JAK2-V617F mutant than in control cells expressing endogenous wild-type JAK2 alone (Figure 2E), thus confirming that JAK2-V617F is more susceptible than wild-type JAK2 to WP1130 in hematopoietic cells as well.

Next, we examined the ubiquitination status of JAK2-V617F induced by WP1130. Kapuria, et al. have demonstrated that WP1130 induced K63-linked polyubiquitination of JAK2 to promote the translocation of the protein into perinuclear aggresomes [23]. As judged by the typical ladder and smeary patterns observed by anti-JAK2 blotting, WP1130 induced polyubiquitination of JAK2-V617F distinctively as compared with wild-type JAK2 transduced into 293T cells along with HA-tagged ubiquitin (Figure 2D). Time-course analyses of JAK2 immunoprecipitates obtained from these cells further revealed that WP1130-induced polyubiquitination of JAK2-V617F took place much faster than that of wild-type JAK2 followed by a more remarkable decrease in its expression level (Figure 2F). It was further revealed that polyubiquitination of JAK2-V617F induced by WP1130 was remarkably reduced by co-transfection of the ubiquitin mutant K63R lacking K63 as compared with wild-type ubiquitin (Figure 2G). On the other hand, co-transfection of K48R mutant lacking K48 caused enhanced polyubiquitination with or without treatment with WP1130. This could be because K48R mutant might have enhanced non-K48-linked types of polyubiquitination, including those with K63-linkage by blocking the most prevalent K48-linkages of ubiquitin chains. As expected, co-transfection of K63R but not K48R retarded WP1130-induced downregulation of JAK2-V617F in these cells (Figure 2G and Figure S2A). 

To examine K63-polyubiquitination of endogenous JAK2-V617F in HEL cells, we then utilized FLAG-K63-TUBE, which specifically binds to K63-linked polyubiquitin chains for immunoprecipitation with anti-FLAG. The specificity of FLAG-K63-TUBE and anti-FLAG to pull down proteins polyubiquitinated through K63 is demonstrated in Figure S2B,C. Treatment with WP1130 or G9 resulted in detection of JAK2-V617F in the immunoprecipitate, which suggests that WP1130 as well as G9 induced K63-linked polyubiquitination of JAK2-V617F or at least its association with proteins polyubiquitinated in this manner in HEL cells (Figure 2H). Taken together, inhibition of USP9X should induce K63-linked, but not K48-linked, polyubiquitination of JAK2-V617F more predominantly than that of wild-type JAK2.

### 2.3. WP1130 Induces Aggresomal Translocation of JAK2 Preferentially for the V617F Mutant Most Likely through Inhibition of USP9X

**Figure 3:**
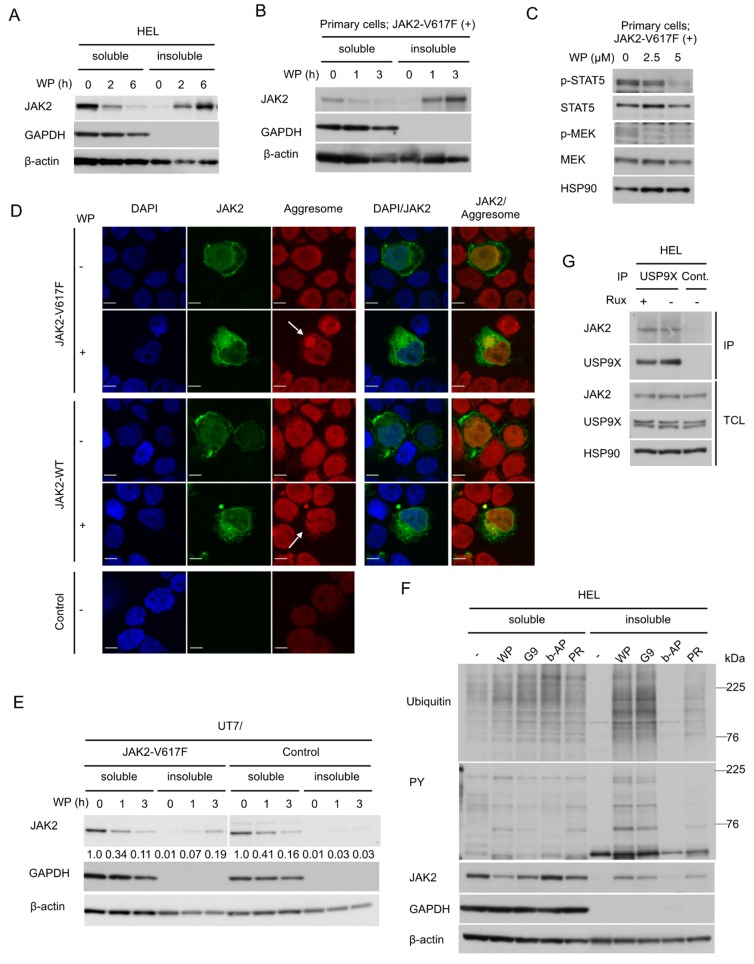
WP1130 induces aggresomal translocation of JAK2 preferentially for the V617F mutant most likely through inhibition of USP9X. (**A**,**B**) HEL (**A**) or primary post-myeloproliferative neoplasms (MPN) secondary acute myeloid leukemic (sAML) cells expressing JAK2-V617F (**B**) were treated with 5 μM WP1130 (WP) for indicated times. Cells were lysed and detergent-soluble and –insoluble proteins were extracted and analyzed. GAPDH and β-actin were used for loading controls and confirmation of appropriate fractionation. (**C**) Primary post-MPN sAML cells expressing JAK2-V617F were treated for 2 h with indicated concentrations of WP1130 and analyzed. HSP90 was used for a loading control. (**D**) 293T cells were transfected with plasmids coding for JAK2-V617F, -WT, or empty vector (Control). Cells were left untreated as control or treated with 5 μM WP1130 for 3 h, followed by processing for confocal microscopy as described in the Materials and Methods. Images represent a 60× optical zoom with a 3× digital zoom. DAPI staining shows the position of the nucleus. Representative images of cells are shown. Positions of aggresomes are indicated by arrows. (**E**) UT7/JAK2-V617F or vector-control cells (Control) were treated with 5 μM WP1130 for indicated times and analyzed. Relative levels of JAK2 as compared with that in cells not treated with WP1130 were determined by densitometric analyses. (**F**) HEL cells were left untreated as control or treated for 3 h with 5 μM WP1130, 3 μM G9, 1 μM b-AP15, or 25 μM PR-619, as indicated. Cells were lysed and detergent-soluble and -insoluble protein were extracted and analyzed. (**G)** HEL cells were treated for 4 h with or without 2 μM ruxolitinib (Rux), as indicated. Immunoprecipitates (IP) with anti-USP9X antibody or normal rabbit IgG (Cont.) and total cell lysate (TCL) were analyzed.

We then examined the aggresomal translocation of JAK2-V617F. As reported for wild-type JAK2 [23], treatment of HEL cells with WP1130 induced a time-dependent decrease of JAK2-V617F in detergent-soluble fraction of cellular proteins accompanied by its reciprocal increase in detergent-insoluble fraction, which is consistent with the aggresomal translocation of JAK2-V617F (Figure 3A). The WP1130-induced translocation of JAK2-V617F was similarly observed in primary leukemic cells (Figure 3B). It was also confirmed that WP1130 inhibited downstream activation of STAT5 and MEK by JAK2-V617F as expected in these cells (Figure 3C). We further confirmed by confocal microscopy that JAK2-V617F (green) accumulated within the perinuclear aggresomes (red) after treatment with WP1130 in 293T cells transfected with JAK2-V617F along with ubiquitin (Figure 3D). The aggresomal translocation was also observed for wild-type JAK2, although it was difficult to quantitatively compare the extent of the translocation with that of JAK2-V617F by these experiments in 293T cells. Thus, we compared the aggresomal translocation of JAK2-V617F and wild type JAK2 in human UT7 cells transduced with JAK2-V617F or a control vector. Downregulation of JAK2 in the soluble fraction was more rapidly observed for JAK2-V617F-expressing cells compared with control cells, which was consistent with observations in 293T and 32D cells (Figure 2D,E and Figure 3E). Furthermore, the aggresomal translocation as judged by increases in the insoluble fraction was distinctively observed for UT7/JAK2-V617F cells but not in control cells, thus supporting the idea that WP1130 more strongly induces the aggresomal translocation of JAK2-V617F than wild-type JAK2. 

To explore the DUB(s) involved in the aggresomal translocation, we treated HEL cells with various DUB inhibitors (Figure 3F). b-AP15 is a proteasomal DUBs inhibitor that blocks USP14 and UCHL5, which are also inhibited by WP1130. PR-619 is a nonselective pan-DUB inhibitor. All the DUB inhibitors examined enhanced ubiquitination of cellular proteins. Importantly, the USP9X inhibitors WP1130 and G9 induced the aggresomal translocation of ubiquitinated and tyrosine-phosphorylated proteins as well as JAK2-V617F. On the other hand, b-AP15 neither downregulated nor induced the aggresomal translocation of these and other ubiquitinated proteins. Pan-DUB inhibitor PR-619 induced the aggresomal translocation of JAK2-V617F to a lesser extent compared with WP1130 or G9. These results indicate that inhibition of USP9X, which is inhibited by WP1130 and G9 in common but not by b-AP15, is most likely responsible for the aggresomal translocation and downregulation of JAK2-V617F. This was further supported by the physical association between JAK2-V617F and USP9X in HEL cells detected by immunoprecipitation assays (Figure 3G). 

### 2.4. WP1130 Induces Apoptosis through Activation of the Intrinsic Mitochondria-Mediated Pathway Synergistically with BH3 Mimetics

**Figure 4:**
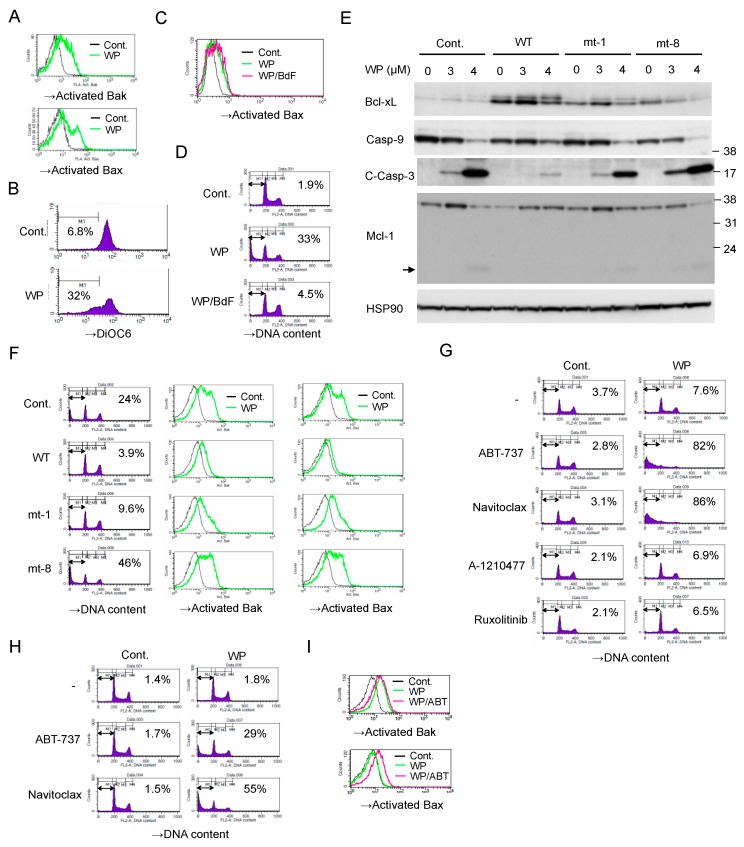
WP1130 induces apoptosis through activation of the intrinsic mitochondria-mediated pathway synergistically with BH3 mimetics. (**A**) HEL cells were left untreated as control (Cont.) or treated with 3 μM WP1130 (WP) for 20 h. Cells were analyzed for activated Bak and Bax by flow cytometry. (**B**) HEL cells were left untreated as control or treated with 3 μM WP1130 for 24 h. Cells were analyzed for the mitochondrial membrane potential (Δφm) by flow cytometry using DiOC6. Percentages of cells with reduced Δφm are indicated. (**C**,**D**) HEL cells were left untreated as control or treated for 24 h with 3 μM WP1130 with or without 100 μM Boc-d-fmk (BdF), as indicated. Cells were analyzed for activation of Bax (**C**) or DNA content (**D**) by flow cytometry. Percentages of apoptotic cells with the sub-G1 DNA content are indicated. (**E**) HEL cells transduced with wild-type (WT) Bcl-xL or its mutants (mt-1 or mt-8) as well as vector control cells (Cont.) were treated with indicated concentrations of WP1130 for 24 h and subjected to immunoblot analysis. C-Casp-3: cleaved Caspase 3. An arrow indicates the putative degradation product of Mcl-1. (**F**) Cells indicated were treated with 3 μM WP1130 for 20 h and analyzed. (**G**) HEL cells were left untreated as control or treated for 24 h with 1 μM ABT-737, 0.5 μM navitoclax, 10 μM A-1210477, or 1μM ruxolitinib with or without 2 μM WP1130, as indicated, and analyzed. (**H**) HEL cells were left untreated as control or treated for 6 h with 1 μM ABT-737 or 1 μM navitoclax with or without 5 μM WP1130, as indicated, and analyzed. (**I**) HEL cells were left untreated as control or treated for 6 h with 1 μM ABT-737 or 3.5 μM WP1130, as indicated, and analyzed.

We next examined the mechanisms involved in induction of apoptosis in JAK2-V617F-expressing cells by WP1130. As shown in Figure 4A,B, WP1130 induced activation of the effector pro-apoptotic Bcl-2 family members Bak and Bax and decreased mitochondrial membrane potential measured by DiOC6 in HEL cells. Pretreatment with the pan-caspase inhibitor Boc-d-FMK did not prevent the WP1130-induced Bax activation, whereas it remarkably inhibited induction of apoptosis as measured by chromosomal DNA fragmentation (Figure 4C,D). These results suggest that WP1130 triggers activation of Bax as well as Bak to induce the mitochondrial membrane depolarization leading to activation of Caspases and Caspase-dependent apoptosis.

We then examined the possible involvement of Bcl-xL and Mcl-1, the major anti-apoptotic Bcl-2 family proteins in hematopoietic cells, in regulation of WP-1130-induced apoptosis in JAK2-V617F-expressing cells. As shown in Figure 4E, WP1130 induced the appearance of a slower migrating form of Bcl-xL, which implies deamidation of Bcl-xL reported to be induced by the DNA damage response and to inactivate its anti-apoptotic function [26,27]. On the other hand, Mcl-1 showed an increase in its expression level in response to WP1130, which, however, was decreased accompanied by an appearance of the plausible degradation fragment when Caspases were drastically activated by a higher concentration of WP1130, which is consistent with our previous report [22]. Overexpression of wild-type Bcl-xL conferred on HEL cells the resistance to WP1130-induced apoptosis as well as Bak and Bax activation (Figure 4E,F). A loss-of-function mutant of Bcl-xL (Bcl-xL mt-8), which binds neither the initiator BH3-only pro-apoptotic Bcl-2 family members tBid, Bim, and Bad, nor the effector members Bak and Bax [28], failed to protect from or even enhanced the apoptosis induced by WP1130 as well as Bak and Bax activation. On the other hand, overexpression of Bcl-xL mt-1, which does not bind the effectors but still inhibits the initiators [28], partly inhibited WP1130-induced apoptosis as well as Bak and Bax activation. These results suggest that the functions of Bcl-xL to prevent activation of both initiator and effector proapoptotic Bcl-2 family members should play important roles in prevention of apoptosis induced by WP1130. 

It was further revealed that the BH3 mimetics ABT-737 and navitoclax, which inhibit Bcl-xL, Bcl-2, and Bcl-w, synergistically and drastically enhanced apoptosis induced by a low concentration of WP1130 in HEL cells (Figure 4G). Moreover, in the presence of ABT-737 or navitoclax, WP1130 was found to induce apoptosis as early as 6 h in HEL cells (Figure 4H). WP1130 activated Bak but not Bax in the absence of BH3-mimetics at this time point (Figure 4I). However, Bax was also activated by WP1130 in the presence of ABT-737, thus suggesting that activation of Bax should be more efficiently protected by the anti-apoptotic Bcl-2 family members and required for induction of apoptosis in these cells. Together, these results are consistent with the idea that inhibition of USP9X activates the intrinsic mitochondria-mediated apoptotic pathway regulated by the intricate balance between pro- and anti-apoptotic Bcl-2 family members. 

### 2.5. WP1130 Causes Oxidative Stress to Activate stress-Related p38/JNK MAPKs Pathways and DNA Damage Responses to Induce Apoptosis

**Figure 5:**
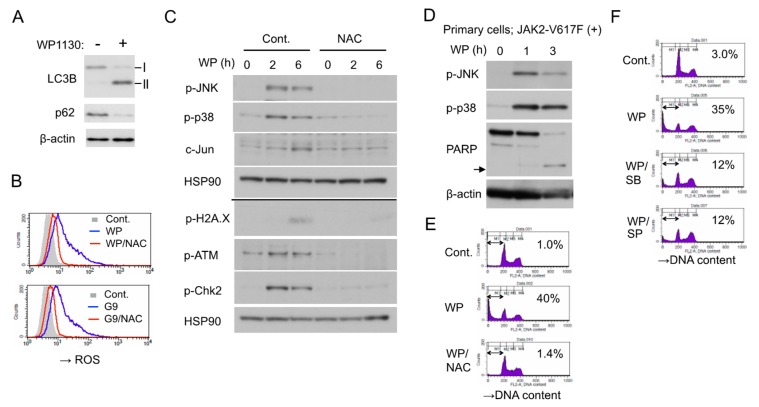
WP1130 causes oxidative stress to activate stress-related p38/JNK MAPKs pathways and DNA damage responses to induce apoptosis. (**A**) HEL cells were treated for 6 h with or without 5 µM WP1130 and subjected to immunoblot analysis with antibodies against indicated proteins. ß-actin was used for a loading control. (**B**) HEL cells were left untreated as control (Cont.) or treated for 3 h with 3 μM WP1130 (WP), 3 μM G9, and 5 mM NAC, as indicated, and analyzed for reactive oxygen species (ROS) by flow cytometry. (**C**) HEL cells left untreated or pretreated with 40 mM NAC for 1 h were further treated with 5 μM WP1130 for indicated times and analyzed by immunoblot analysis. The results obtained from duplicate gels are shown above or below a thin horizontal line. (**D**) Primary post-MPN sAML cells expressing JAK2-V617F were treated with 5 μM WP1130 for indicated times and analyzed. An arrow indicates the position of cleaved PARP. (**E**) HEL cells were left untreated as control or treated for 24 h with 3.5 μM WP1130 and 5 mM NAC, as indicated, and analyzed for DNA content. Percentages of apoptotic cells with the sub-G1 DNA content are indicated. (**F**) HEL cells were left untreated as control or treated for 24 h with 3.5 μM WP1130, 50 μM SB203580, and 50 μM SP600125, as indicated, and analyzed.

WP1130 induced apoptosis in HEL cells along with downregulation of JAK2-V617F and downstream signaling. However, inhibition of JAK2-V617F using ruxolitinib or other JAK inhibitors alone failed to induce apoptosis in HEL cells as shown in Figure 4G and as reported previously [5], indicating that blockage of JAK2 signaling was not sufficient for apoptosis induction by itself. In this regard, we previously demonstrated that inhibition of JAK2-V617F induces autophagy to prevent apoptosis in leukemic cells including HEL [5], while WP1130 was reported to block autophagy by inducing the aggresomal translocation and inhibition of UKL1 [29]. Nevertheless, WP1130 increased autophagy in HEL cells, as judged by an increased or decreased expression of LC3-II or p62/SQSTM1 (Figure 5A). On the other hand, we have recently revealed that inhibition of USP9X by WP1130 or G9 induced oxidative stress to stimulate stress-related MAP kinase pathways and DNA damage responses, which cooperated with inhibition of FLT3-ITD signaling to induce apoptosis efficiently in FLT3-ITD-positive AML cells [22]. Thus, we next examined whether similar responses are induced by inhibition of USP9X in HEL cells. As expected, WP1130 or G9 rapidly increased the intracellular reactive oxygen species (ROS) levels, which was mostly prevented by cotreatment with the ROS scavenger N-acetyl-L-cysteine (NAC) (Figure 5B) Furthermore, WP1130 activated stress-related p38 as well as JNK, leading to upregulation of c-Jun expression and induced DNA damage responses, including phosphorylation of histone H2A.X, ATM, and Chk2 (Figure 5C). It was confirmed that WP1130 induced activation of p38 as well as JNK and apoptosis, as judged by PARP cleavage, also in the JAK2-V617F-positive primary leukemic cells (Figure 5D). It was further revealed that inhibition of increases in ROS by NAC prevented the WP1130-induced activation of JNK as well as p38, DNA damage responses, and apoptosis, while pharmacological inhibition of p38 by SB203580 or JNK by SP600125 attenuated WP1130-induced apoptosis in HEL cells (Figure 5C,E,F). It is thus suggested that the WP1130-induced oxidative stress leading to activation of stress-related MAP kinases and DNA damage responses is required for induction of apoptosis in these cells along with the blockage of signaling from JAK2-V617F.

### 2.6. Ruxolitinib-persistent HEL-R Cells Exhibit an Increased Sensitivity to WP1130 or BH3 Mimetics

**Figure 6:**
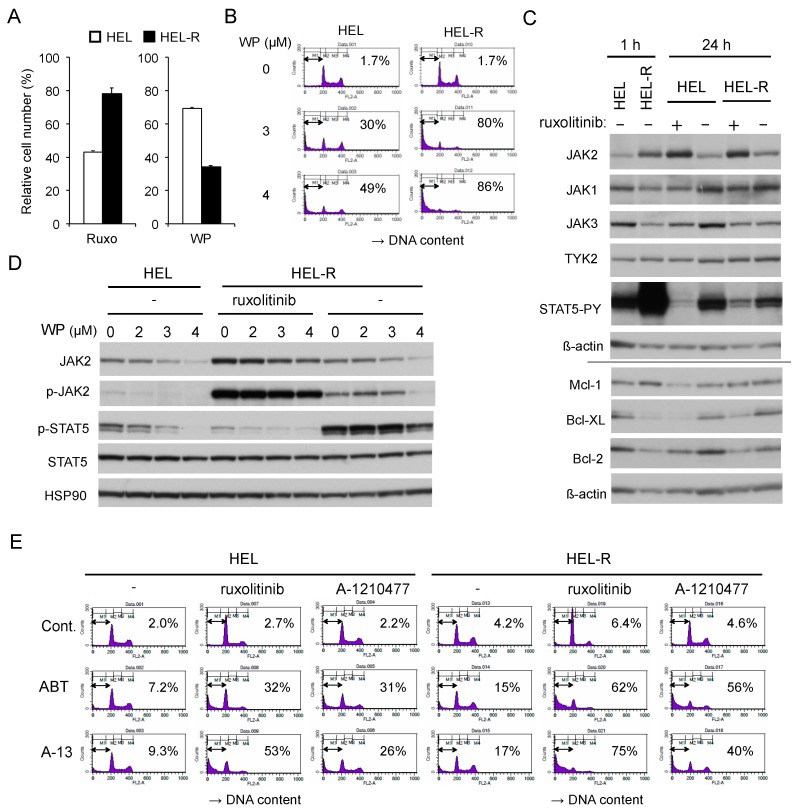
Ruxolitinib-persistent HEL-R cells exhibit an increased sensitivity to WP1130 or BH3 mimetics as compared with control HEL cells. (**A**) HEL or ruxolitinib-persistent HEL-R cells were left untreated as control or treated with 1 μM ruxolitinib for 48 h or with 2 μM WP1130 for 24 h. Viable cell numbers were measured by the CCK-8 colorimetric assay. Each column represents the mean of triplicate cultures, with error bars indicating standard errors, and is expressed as a ratio to the control. (**B**) HEL or HEL-R cells were treated with indicated concentrations of WP1130 for 24 h. Cells were analyzed for DNA content by flow cytometry. Percentages of apoptotic cells with the sub-G1 DNA content are indicated. (**C**) HEL or HEL-R cells were washed and cultured with or without 1 µM ruxolitinib for indicated times and lysed for immunoblot analysis with antibodies against indicated proteins. The results obtained from duplicate gels are shown above or below a horizontal line. ß-actin was used for a loading control. (**D**) HEL or HEL-R cells were treated for 3 h with indicated concentrations of WP1130 with or without 1 μM ruxolitinib, as indicated, and analyzed. HSP90 was used for a loading control. (**E**) HEL or HEL-R cells were left untreated as control or treated for 40 h with 1 μM ABT-737 (ABT), 5 nM A-1331852 (A-13), 1 μM ruxolitinib, or 5 μM A-1210477, as indicated, and analyzed.

Finally, we examined the efficacy of WP1130 on HEL cells under the conditions of ruxolitinib persistence. We cultured HEL cells with increasing concentration of ruxolitinib in the long term, essentially as described previously [12], to develop HEL-R cells, whose proliferation was affected less remarkably by ruxolitinib than naive HEL cells that had been cultured without ruxolitinib (Figure 6A). Interestingly, HEL-R cells were much more susceptible to WP1130 in terms of both inhibition of proliferation and induction of apoptosis in the presence or absence of ruxolitinib (Figure 6A,B and Figure S3A,B). In the presence of ruxolitinib or shortly after its removal, JAK2-V617F, but not other JAK family kinases, was expressed at a remarkably higher level in HEL-R cells than in naive HEL cells in accordance with the previous report (Figure 6C) [12]. The level of JAK2-V617F in naive HEL cells or in HEL-R cells increased or decreased remarkably in 24 h after adding or removing ruxolitinib, respectively, which was in contrast to the other JAK family members. In accordance with previous reports [12,30], JAK2-V617F was transiently hyperactivated when HEL-R cells were washed out from ruxolitinib, resulting in a drastic hyperphosphorylation of STAT5. On the other hand, under the ruxolitinib persistence condition, STAT5 was phosphorylated in HEL-R cells at a more reduced level than in naive HEL cells cultured without ruxolitinib (Figure 6C,D). In HEL-R cells, JAK2-V617F, and its phosphorylated form in particular, distinctively showed the resistance for WP1130-induced downregulation, especially in the absence of ruxolitinib as compared with those in naive HEL cells (Figure 6D). Consistently, activation-specific phosphorylation of STAT5, which was reduced or drastically enhanced in HEL-R cells in the presence or absence of ruxolitinib, respectively, partially showed the resistance to WP1130 for suppression. Thus, the increase in susceptibility of HEL-R cells to WP1130 for inhibition of proliferation as well as induction of apoptosis should be caused by mechanisms not related to WP1130-induced downregulation of JAK2-V617F or its downstream signaling. 

To explore the possible mechanisms involved in the increase in sensitivity of HEL-R cells to WP1130, we next examined expression levels of anti-apoptotic Bcl-2 family proteins. As shown in Figure 6C, expression levels of Bcl-2 and Bcl-xL, but not that of Mcl-1, were decreased in HEL-R cells under the ruxolitinib persistence condition as compared with those in ruxolitinib-naive HEL cells, while they increased or decreased by withdrawal from or addition of ruxolitinib, respectively, for 24 h in these cells in parallel with phosphorylation levels of STAT5. Furthermore, HEL-R cells showed increased sensitivities to ABT-737 or the selective Bcl-xL inhibitor A-1331852 for induction of apoptosis compared with control cells (Figure 6E). Ruxolitinib or the Mcl-1 inhibitor A-1210477 hardly induced apoptosis in HEL-R cells as well as in naive HEL cells in accordance with our previous report [5]. However, these inhibitors synergistically enhanced apoptosis induced by ABT-737 or A-1331852, which was also more remarkably observed in HEL-R cells. As expected, the combined treatment with WP1130 and ABT-737 induced apoptosis also more drastically in HEL-R cells than in control HEL cells in the absence or presence of ruxolitinib (Figure S3C). These results suggest that ruxolitinib-persistent HEL-R cells show enhanced sensitivity to WP1130 at least partly due to the decreased expression of Bcl-2 and Bcl-xL.

## 3. Discussion

Our study has extended the previous study by Kapuria, et al. [23] by revealing that WP1130 or G9 downregulates JAK2-V617F more efficiently than wild-type JAK2. Thus, unlike the JAK1/2 inhibitor ruxolitinib currently in clinical use, these USP9X inhibitors more efficiently affected hematopoietic cells dependent on JAK2-V617F than on cytokine, which bears an important clinical implication for the possible future therapeutic application of USP9X inhibition for MPNs with this mutation. The difference in sensitivity is most likely because these DUB inhibitors preferentially affect the activated conformation of JAK2 autophosphorylated on Y1007/1008. In this regard, we have previously reported that Epo-induced autophosphorylation of JAK2 on Y1007/1008 promoted polyubiquitination by the RING finger family E3 ligases c-Cbl and Cbl-b leading to proteasomal degradation of JAK2 [31], which is consistent with previous studies revealing that activated kinases are downregulated via the ubiquitin/proteasome pathway [14]. Therefore, it is expected that the activated mutant JAK2-V617F is more prone to ubiquitination than wild-type JAK2, which should at least partly explain the difference in sensitivity to the DUB inhibitor WP1130. In fact, we revealed that JAK2-V617F was more prominently polyubiquitinated than wild-type JAK2 with or without treatment with WP1130 when heterogeneously expressed in 293T cells (Figure 2F). It should be noted, however, that WP1130 enhanced mainly K63-mediated but not K48-mediated polyubiquitination of JAK2-V617F in 293T cells (Figure 2G), which is concordant with the previous study on wild-type JAK2 [23]. Moreover, enhancement of polyubiquitination of JAK2-V617F or wild-type JAK2 by WP1130 led to the aggresomal translocation but not the proteasomal degradation, which is dependent mainly on K48-linked polyubiquitination. On the other hand, Liu et al. [32] have recently reported that c-Cbl induced K63-linked polyubiquitination of JAK2 at K970 to enhance JAK2 activation and downstream signaling upon granulocyte-macrophage (GM)-CSF stimulation in HeLa cells transduced with these molecules. We speculate that the reconstitution of the GM-CSF receptor/JAK2 system in the nonhematopoietic cell line might have caused the apparently conflicting effects of K63-linked polyubiquitination on JAK2 observed between the study by Liu et al. [32] and ours as well as that by Kapuria, et al. [23]. Furthermore, our previous study showed that c-Cbl and Cbl-b induced K48-linked polyubiquitination of autophosphorylated FLT3-ITD as well as JAK2-V617F, while the HECT family E3 ligase NEDD4 was implicated in K63-linked polyubiquitination of FLT3-ITD [31,33], which underwent the aggresomal translocation similarly with JAK2-V617F when treated with WP1130 [22]. Thus, future studies are required to identify the E3 ligase responsible for K63-linked polyubiquitination of JAK2-V617F as well as to define the possible roles of c-Cbl and the K63-specific ligase NEDD4 [34] in this process.

Although WP1130 or G9 inhibits several DUBs, the comparison of specificities of various DUB inhibitors utilized and their effects on JAK2-V617F (Figure 3F) strongly suggests that inhibition of USP9X should be mainly responsible for the aggresomal translocation of JAK2-V617F. The involvement of USP9X in deubiquitination of JAK2-V617F is further supported by the physical association between endogenous USP9X and JAK2-V617F observed in HEL cells (Figure 3G), which extends the previous study by Chou et al. [35] demonstrating this association in a transfection experiment in a nonhematopoietic cell line. Furthermore, Chou et al. [35] demonstrated that cytokine-activated JAK2 signaling was inhibited by BRD0476, which is more specific for USP9X than WP1130, or by knockout of USP9X. Although the cellular localization was not examined, they showed by ELISA that BRD0476 decreased the expression level of JAK2 phosphorylated on Y1007/1008 but not that of total JAK2. Moreover, based on data obtained by site-directed mutagenesis of JAK2 and on the inability to detect any effect of cytokines or the USP9X inhibitor on the overall ubiquitination status of JAK2, they speculated that JAK2 might be monoubiquitinated on K999, which was expected to inhibit activation of JAK2 and to be deubiquitinated by USP9X. The discrepancies between the study by Chou et al. [35] and ours as well as that by Kapuria, et al. [23] may be explained by the differences in cellular contexts (pancreatic ß-cells undergoing apoptosis upon stimulation by the inflammatory cytokine interferon-gamma vs. hematopoietic cells induced to proliferate upon stimulation by the hematopoietic cytokines IL-3 and Epo or by the JAK2-V617F mutant) or by the different experimental methods (ELISA of RIPA lysates vs. cellular fractionation and confocal immunofluorescent microscopy). In this regard, it should be noted that the present study has confirmed that WP1130 induced the aggresomal translocation of JAK2-V617F also in primary post-MPN sAML cells. Although the underlying mechanisms were not shown, a recent study demonstrated that the USP9X inhibitor BRD0476 downregulated JAK2 signaling also in a subtype of acute lymphoblastic leukemia (ALL) that shows hyperactivation of JAK2 with or without the activating R683G mutation [36]. For these ALL cells, hyperactivation of JAK signaling may not be tolerable, because BRD0476 or low-dose ruxolitinib enhanced the survival of these cells. Intriguingly, USP9X was frequently mutated or deleted in this subtype of ALL, thus further supporting the close relationship between USP9X and JAK2. It should be also noted, however, that G9, as well as WP1130, was recently reported to inhibit USP24 as well as USP5 in addition to USP9X [37]. Thus, the possible involvement of USP24 or USP5, which is not affected by b-AP, needs to be addressed in future studies. 

WP1130 not only blocked JAK2-V617F signaling but also induced oxidative stress signaling, leading to DNA damage responses, which was required to induce apoptosis rapidly and efficiently in HEL cells as judged by the effects of NAC or inhibitors for JNK and p38 (Figure 5). This is in accordance with our previous data that HEL cells hardly undergo apoptosis by inhibition of JAK2-V617F alone [5], which may partly explain the limited efficacy of ruxolitinib in eradicating the JAK2-V617F clone in both MPNs and post-MPN secondary AML [8,9,10]. Although the generation of ROS independent of the mitochondria plays important roles in cytokine-induced activation of JAK2 and its downstream signaling pathways [38], ROS accumulated by JAK2-V617F in cells results in DNA damages and genomic instability leading to disease progression [39,40]. Elevated intracellular ROS levels have been observed also in other hematological malignancies, and treatment with pro-oxidants to amplify the pre-existent oxidative stress is supposed to cause catastrophic damages in these cells [41]. It is thus plausible that WP1130 increases intracellular ROS to the levels that exceed the cellular anti-oxidant capacity to activate stress-induced signaling and subsequent apoptotic pathways very efficiently. However, although the WP1130-induced increase in ROS levels was observed also in the FLT3-ITD-positive AML cell line MV4-11 [22], it was not observed in CML and mantle cell lymphoma cell lines [16,18], thus suggesting that it may be dependent on cellular contexts. The mechanisms involved in ROS production induced by WP1130 have remained unknown. In this regard, DUBs are involved in quality control of the mitochondria, disturbance of which increases ROS generation leading to oxidative damage [42]. Thus, it is plausible that WP1130 may increase the ROS generation by disturbing the mitochondria through inhibition of DUBs, including USP9X known to be present at least partly at the mitochondria [36,43]. Future studies are required to elucidate the exact mechanisms involving the ROS generation by inhibition of USP9X and underlying the activation of the intrinsic apoptotic pathway cooperatively by increased ROS and inhibition of JAK2-V617F. 

WP1130 induces apoptosis through the intrinsic mitochondrial pathway because it first activated Bak and Bax followed by the decrease in mitochondrial membrane potential leading to activation of Caspases-9 and -3. Furthermore, activation of Bax was not inhibited by the pan-caspase inhibitor Boc-d-FMK but reduced by overexpression of the Bcl-xL mutant mt-1, which inhibits the initiator BH3-only pro-apoptotic Bcl-2 family members tBid, Bim, and Bad, but not Bak and Bax [28], thus indicating the involvement of these initiators in activation of the apoptotic pathway. Intriguingly, activation of Bak was induced by WP1130 very rapidly, while that of Bax took place later or was accelerated by inhibition of the anti-apoptotic Bcl-2 family members and accompanied by induction of apoptosis. Thus, it is speculated that Bax activation should be required for execution of apoptosis and may be dependent on downregulation of the anti-apoptotic Bcl-2 family members. In this regard, WP1130 increased rather than decreased Mcl-1 expression until caspases were fully activated, while it induced the appearance of a slower migrating species of Bcl-xL in Western blot analysis (Figure 4E). Although Mcl-1 expression was reduced ultimately in accordance with various previous reports [43], it was observed only when caspases were fully activated and was most likely through cleavage by caspases, thus generating the plausible cleaved fragment (Figure 4E). This is inconsistent with previous reports implicating proteasomal degradation as the molecular mechanism involved in Mcl-1 downregulation by USP9X inhibition [43,44]. However, inactivation of Mcl-1 or its conversion to a pro-apoptotic protein by the cleavage by caspases has also been reported mainly in hematopoietic cells [45,46], and we have previously demonstrated that WP1130 decreased Mcl-1 expression through the cleavage by caspases also in FLT3-ITD-positive AML cells [22]. On the other hand, the WP1130-induced appearance of a slower migrating species of Bcl-xL was observed even when activation of caspases was prevented by overexpression of Bcl-xL (Figure 4E), which implies that Bcl-xL was deamidated. The deamidation of Bcl-xL has been reported to be induced in response to various DNA-damaging agents and to inactivate or destabilize Bcl-xL through various mechanisms dependent on cellular contexts [47]. Intriguingly, JAK2-V617F or BCR/ABL has been reported to inhibit deamidation of Bcl-xL in response to DNA damage through mechanisms involving activation of the PI3K/Akt pathway to increase ROS levels and probably through other mechanisms not well defined [27,39,47]. Thus, it is plausible that, by both causing DNA damage responses and inhibiting JAK2-V617F signaling, WP1130 induced deamidation of Bcl-xL to downregulate its anti-apoptotic function.

The present study has revealed that, as compared with control naive HEL cells, ruxolitinib-persistent HEL-R cells showed increased sensitivities to WP1130 for inhibition of proliferation and induction of apoptosis, although JAK2-V617F, including that phosphorylated on Y1007/1008, was distinctively resistant to WP1130-induced downregulation as compared with that in control cells (Figure 6). In HEL-R cells, JAK2-V617F was expressed at a remarkably increased level and strongly phosphorylated in the presence of ruxolitinib in accordance with previous reports [12,30,48,49]. Previously, it was proposed that JAK1 and TYK2 phosphorylated JAK2-V617F to activate its kinase activity under the ruxolitinib persistence conditions, mostly because JAK2-V617F overexpressed and hyperphosphorylated in ruxolitinib-persistent SET-2 and UKE-1 cells was co-immunoprecipitated with these JAK family kinases [12]. However, we failed to demonstrate the physical association of JAK2-V617F with these kinases in HEL-R cells in contrast to the previous study, in which the association was demonstrated even in ruxolitinib-naive SET-2 and UKE-1 cells [12]. More recently, it has been reported that ruxolitinib induces accumulation of tyrosine-phosphorylated JAK2 by binding to its activated form as the type I JAK inhibitor and changing its conformation to prevent dephosphorylation as well as ubiquitination required for degradation [30,48,49]. Thus, tyrosine-phosphorylated JAK2-V617F accumulates in the presence of ruxolitinib in the intermediate active kinase conformation [49], which leads to a drastic activation of JAK2-V617F upon removal of ruxolitinib to cause the severe withdrawal syndrome observed specifically in JAK2-V617F-positive MPN patients with abruptly discontinued ruxolitinib [30]. It is also anticipated that inhibition of DUBs, including USP9X by WP1130, may affect JAK2-V617F phosphorylated and bound to ruxolitinib in HEL-R cells less significantly than JAK2-V617F in naive HEL cells. Although JAK2-V617F was transiently hyperactivated and induced a drastic activation of STAT5 when HEL-R cells were washed out from ruxolitinib in accordance with previous reports [12,30], STAT5 was activated only weakly under the ruxolitinib persistence condition (Figure 6C). Consistent with this, HEL-R cells showed decreased expression levels of Bcl-xL and Bcl-2, which are anti-apoptotic Bcl-2 family proteins that are regulated at least partly through the JAK/STAT pathway at the transcription level and play major roles in the resistance of JAK2-V617F-driven cells against JAK inhibitors [5,50,51,52]. This is in part consistent with the previous report by Waibel, M. et al. [51], which demonstrated a low expression level of Bcl-2 and an increased transcription level of the Bcl-xL gene induced by hyperactivated STAT5 in the ruxolitinib-persistent cells washed out from the inhibitor. Although future studies are needed to more fully define the molecular mechanisms underlying the reduced expression of Bcl-2 and Bcl-xL in HEL-R cells, the present study has revealed that these cells also showed increased sensitivities to BH3 mimetics inhibiting Bcl-xL as well as Bcl-2 for induction of apoptosis. This supports the relevance of decreased expression of these proteins for the increased susceptibility of HEL-R to WP1130-induced apoptosis. Therefore, the present study suggests USP9X, as well as the anti-apoptotic Bcl-2 members, as a promising target for novel therapeutic strategies against JAK2-V617F-positive MPNs with the ruxolitinib resistance.

## 4. Materials and Methods 

### 4.1. Cells

HEL and K562 were obtained from Fujisaki Cell Center (Okayama, Japan) and the Riken Cell Bank (Ibaraki, Japan), respectively. The JAK2-V617F-expressing post-MPN sAML cell line PVTL-2 was previously established in our laboratory as reported previously [5]. These cells were cultured in RPMI1640 medium with 10% fetal calf serum (FCS). 32DE, murine hematopoietic 32Dcl3 cells expressing the heterogenous Epo receptor, was previously described [24] and cultured in RPMI1640 medium with 10% FCS and 0.5 U/ml human recombinant Epo. 32DE cells expressing the JAK2-V617F mutant, 32DE/JAK2-V617F, have been described previously [53]. The human leukemic cell line expressing the endogenous Epo receptor UT7 [54] was kindly provided by Dr. N. Komatsu and maintained in RPMI1640 medium with 10% FCS and 0.5 U/ mL human recombinant Epo. UT7 cells expressing the JAK2-V617F mutant, UT7/JAK2-V617F, have been described previously [53]. HEL cells overexpressing Bcl-xL or its mutants have been described previously [5]. Ruxolitinib-persistent HEL cells, HEL-R, were obtained by culturing HEL cells with increasing concentrations of ruxolitinib starting from 0.5 µM up to 1.0 µM for more than 6 weeks, essentially as described previously [12]. PLAT-A, an amphotropic virus packaging cell line, and 293T, a human embryonic kidney cell line, were kindly provided by Drs. T. Kitamura and S. Yamaoka, respectively, and maintained in Dulbecco’s modified Eagle’s medium (DMEM) supplemented with 10% FCS. 

### 4.2. Reagents and Antibodies

Deubiquitinase inhibitors WP1130, G9, b-AP15, and PR-619 were purchased from EMD Chemicals (San Diego, CA, USA), Aobious (Gloucester, MA, USA), Cayman Chemical (Ann Arbor, MI, USA), and Focus Biomolecules (Plymouth Meeting, PA, USA), respectively. The JAK1/2 inhibitor ruxolitinib and the pan-caspase inhibitor Boc-D-FMK were purchased from LC Laboratories (Boston, MA, USA) and BioVision (Milpitas, CA, USA), respectively. Inhibitors for p38, SB203580, and for JNK, SP600125, were purchased from Merk Millipore (Darmstadt, Germany) and Santa Cruz Biotechnology (Dallas, TX, USA), respectively. BH3-mimetics ABT-737 and navitoclax (ABT-263) were purchased from Selleck Chemicals (Houston, TX, USA) and AdooQ BioScience (Irvine, CA, USA), respectively. BH3 mimetics A-1210477 and A-1331852 were purchased from Chemietek (Indianapolis, IN, USA). Propidium iodide (PI), doxycycline, goat serum, and N-Acetyl-L-Cysteine (NAC) were purchased from Sigma-Aldrich (St Louis, MO, USA). 3,3‘-Dihexyloxacarbocyamine iodide (DiOC6) and puromycin were purchased from Invitrogen (Carlsbad, CA, USA) and Clontech (Mountain View, CA, USA), respectively. Recombinant human Epo was kindly provided by Chugai Pharmaceutical (Tokyo, Japan). 

For immunoblot analysis, monoclonal antibodies against phospho-JAK2 (Tyr1007/1008) (#3776), JAK2 (#3230), phospho-STAT5 (Tyr694) (#9359), phospho-MEK1/2 (Ser217/221) (#9121), phospho-p90RSK (Ser380) (#9335), phospho-p70 S6 Kinase (Thr389) (#9234), phospho-4EBP1 (Thr37/46) (#2855), MEK1/2 (#9122), HA-Tag (#3724), phospho-SAPK/JNK (Thr183/Tyr185) (#9251), phospho-p38 MAP Kinase (Thr180/Tyr182) (#9211), phospho-Histone H2A.X (Ser139) (#9718), phospho-ATM (Ser1981) (#4526), phospho-Chk2 (Thr68) (#2661), JAK1 (#3344), JAK3 (#8827), Bcl-xL (#2461), Mcl-1 (#5453), and LC3B (#2775) were purchased from Cell Signaling Technology (Danvers, MA, USA). Antibodies against HSP90 (sc-13119), GAPDH (sc-32233), Ubiquitin (sc-8017), c-Jun (sc-44), and Bcl-2 (sc-783) were purchased from Santa Cruz Biotechnology. Antibodies against β-actin (A1978) and phosphotyrosine (05-321) were purchased from Sigma-Aldrich and Merck Millipore, respectively. Antibodies against TYK2 (TL-20220) and Poly (ADP-ribose) polymerase (PARP) (SA-250), as well as anti-Ubi-K63 (BML-PW0600), were purchased from BD Biosciences (San Jose, CA, USA) and Enzo Life Sciences (Farmingdale, NY, USA), respectively. Antibody against p62/SQSTM1 was purchased from Medical and Biological Laboratories (Nagoya, Japan). For immunoprecipitation assay, polyclonal antibodies against JAK2 (06-255-I), USP9x (55054-1-AP), and normal rabbit IgG (#2729) as a control were purchased from Merck Millipore, Proteintech (Rosemont, IL, USA), and Cell Signaling Technology, respectively. Polyclonal anti-USP9X was also used for immunoblot analysis. For flow cytometric analysis, conformation-specific monoclonal antibodies against activated Bak (Ab-1) (AM03) and activated Bax (YTH-6A7) (2281-MC-100) were purchased from Merck Millipore and Trevigen (Gaithersburg, MD, USA), respectively, and anti-mouse F(ab’)2 IgG (H+L) APC-conjugated antibody (F0101B) was purchased from R&D Systems (Minneapolis, MN, USA). For immunocytochemistry, monoclonal anti-JAK2 (Cell Signaling Technology, #3230) was used as a primary antibody, and anti-rabbit F(ab’)2 IgG (H+L) Alexa Flour® 647-conjugated antibody (Cell Signaling Technology, #4414) was used as a secondary antibody.

### 4.3. Expression Plasmids, Transfection, and Infection

Retroviral expression plasmids for JAK2 and the JAK2-V617F mutant (pRev-TRE-JAK2 and pRev-TRE-JAK2-V617F) were described previously [7,31]. Retroviral vectors, pRK5-HA-Ubiquitin-WT and -K48R, were gifts from Ted Dawson (Addgene plasmid #17608 and #17604, respectively) [55]. A retroviral vector pRK5-HA-Ubiquitin-K63R [56] was kindly provided by Dr. J. Held. A lentiviral packaging plasmid psPAX2 and an envelope expressing plasmid pMD2.G were gifts from Didier Trono (Addgene plasmid #12260 and #12259, respectively). A control lentiviral expression vector, Luciferase-pcw107, was a gift from David Sabatini and Kris Wood (Addgene plasmids #64648) [57]. Retroviral vectors, pRevTet-ON and pRxZiN, were obtained from Clontech and the Riken Gene Bank (Ibaraki, Japan), respectively.

For transient expression in 293T cells, cells were transfected with pRevTRE-JAK2 (-WT or -V617F), pRK5-HA-Ubiquitin (-WT, -K48R, or -K63R), and pRevTet-ON using the Lipofectamine reagent (Invitrogen) according to the manufacturer’s instructions. After transfection, cells were further cultured for 1 day with 1 µg/ml doxycycline to induce JAK2 expression before harvest. To obtain vector control cells for 32DE/JAK2-V617F, 32DE cells were infected with the recombinant retrovirus obtained from PLAT-A transfected with the pRxZiN empty vector followed by selection with G418 (Wako, Osaka, Japan). To obtain vector control cells for UT7/JAK2-V617F, UT7 cells were infected with the recombinant lentivirus obtained from 293T transfected with Luciferase-pcw107, psPAX2, and pMD2.G. Cells were selected with 1 μg/ml puromycin.

### 4.4. Cell Proliferation and Flow Cytometric Analyses

Cell proliferation was assessed by Cell counting kit-8 (CCK-8) (Dojindo molecular technologies, Kumamoto, Japan) as previously described [22]. Flow cytometric analyses of apoptosis, the mitochondrial membrane potential, activation of Bak and Bax, and the intracellular ROS levels were performed as previously described [22,58]. For flow cytometric analysis of the mitochondrial membrane potential, cells were incubated with DiOC6 - PI staining solution (60 nM DiOC6, 5 µg/mL PI, 0.2% BSA in PBS) for 15 min at 37 °C and analyzed. All of the flow cytometric analyses were performed using BD FACSCalibur (BD Biosciences) and analyzed with BD CellQuest Pro software (BD Biosciences).

### 4.5. Immunocytochemistry

Immunocytochemistry of 293T cells was performed essentially as previously described [22]. In brief, cells centrifuged onto slides were fixed in 4% paraformaldehyde and permeabilized in 0.1% Triton X-100 for 10 min each. Slides were then incubated stepwise in blocking solution (5% goat serum in PBS), the primary antibody (1:200 in the blocking solution), and then the secondary antibody (1:1000 in the blocking solution) for 1 h each at room temperature. The aggresomes were visualized using PROTEOSTAT Aggresome Detection Kit (Enzo Life Sciences), and the nucleus was counterstained using ProLong Gold antifade reagent with DAPI (Invitrogen). Images were acquired using a confocal microscope (Fluoview FV10i-DOC, Olympus, Tokyo, Japan) and analyzed with FV10 ASW10 software (Olympus).

### 4.6. Immunoprecipitation and Immunoblotting

For immunoprecipitation and immunoblotting experiments, cells were lysed and analyzed, essentially as described previously [22]. Immunoblotting experiments of detergent-soluble total cell lysate and -insoluble fraction and immunoprecipitation assay including K63-Ubiquitin pull-down assay with FLAG K63-TUBEs (Tandem Ubiquitin Binding Entities) (LifeSensors, Malvern, PA, USA) were performed as previously described [22]. 

### 4.7. Analyses of Primary Cells

Primary leukemic cells from a patient with JAK2-V617F-positive post-PMF AML were analyzed essentially as described previously [59]. The allele burden of JAK2-617F was 38.3%, and the peripheral blood containing 30% or 43% blasts was used to isolate mononuclear cells for analyses. The study was approved by the ethical committee of Tokyo Medical and Dental University. Written informed consent was obtained from the patient in compliance with the Declaration of Helsinki.

### 4.8. Statistical Analysis

An unpaired two-tailed Student’s t-test was used to calculate differences between means of two samples. One-way ANOVA test was used to calculate differences between means of three samples, followed by post-hoc analysis using Dunnett’s test to compare each sample as indicated. All statistical analyses were performed with EZR (Saitama Medical Center, Jichi Medical University, Saitama, Japan), a graphical user interface for R (The R Foundation for Statistical Computing, Vienna, Austria).

## 5. Conclusions

We have revealed for the first time that the USP9X inhibitor WP1130 or G9 enhances the K63-linked polyubiquitination and aggresomal translocation of autophosphorylated JAK2-V617F more remarkably than cytokine-activated wild-type JAK2 to preferentially affect cells dependent on this mutant, including leukemic cells. Induction of apoptosis by inhibition on USP9X was mediated through the intrinsic mitochondrial pathway and was enhanced synergistically by BH3 mimetics. Furthermore, WP1130 or G9 induced oxidative stress to activate stress-related MAP kinases p38 and JNK as well as DNA damage responses, which was required for induction of apoptosis. Importantly, as compared with ruxolitinib naive HEL cells, WP1130 more significantly affected the ruxolitinib-resistant JAK2-V617F-positive HEL-R cells under the ruxolitinib persistence condition, which expressed Bcl-xL and Bcl-2 at reduced levels and showed increased sensitivities to BH3 mimetics inhibiting these anti-apoptotic molecules. Thus, the present study proposes USP9X inhibitors along with BH3 mimetics as promising agents for novel therapeutic strategies against JAK2-V617F-positive myeloproliferative neoplasms, particularly under the ruxolitinib persistence condition.

## Supplementary Materials

The following are available online at https://www.mdpi.com/2072-6694/12/2/406/s1. Figure S1: WP1130 preferentially enhances K63-linked polyubiquitination and downregulates JAK2-V617F; Figure S2: ruxolitinib-persistent HEL-R cells exhibit an increased sensitivity to WP1130 in the presence of ruxolitinib as compared with control HEL cells; Figure S3: Ruxolitinib persistent HEL-R cells exhibit an increased sensitivity to WP1130 in the presence of ruxolitinib as compared with control HEL cells.
