# Supplementary material for: Inhibition of USP9X Downregulates JAK2-V617F and Induces Apoptosis Synergistically with BH3 Mimetics Preferentially in Ruxolitinib-Persistent JAK2-V617F-Positive Leukemic Cells
Source: Cancers (Basel). 2020 Feb 10;12(2):406. doi: 10.3390/cancers12020406 (PMC7072561; doi:10.3390/cancers12020406)
Supplement: Supplementary file 1 [file cancers-12-00406-s001.zip › cancers-702627-suppl-final/cancers-702627-suppl-final.docx]

Article

Inhibition of USP9X Downregulates JAK2-V617F and Induces Apoptosis Synergistically with BH3 Mimetics Preferentially in Ruxolitinib-Persistent JAK2-V617F-Positive Leukemic Cells

Hiroki Akiyama, Yoshihiro Umezawa, Daisuke Watanabe, Keigo Okada, Shinya Ishida, Ayako Nogami and Osamu Miura

Supplementary Materials


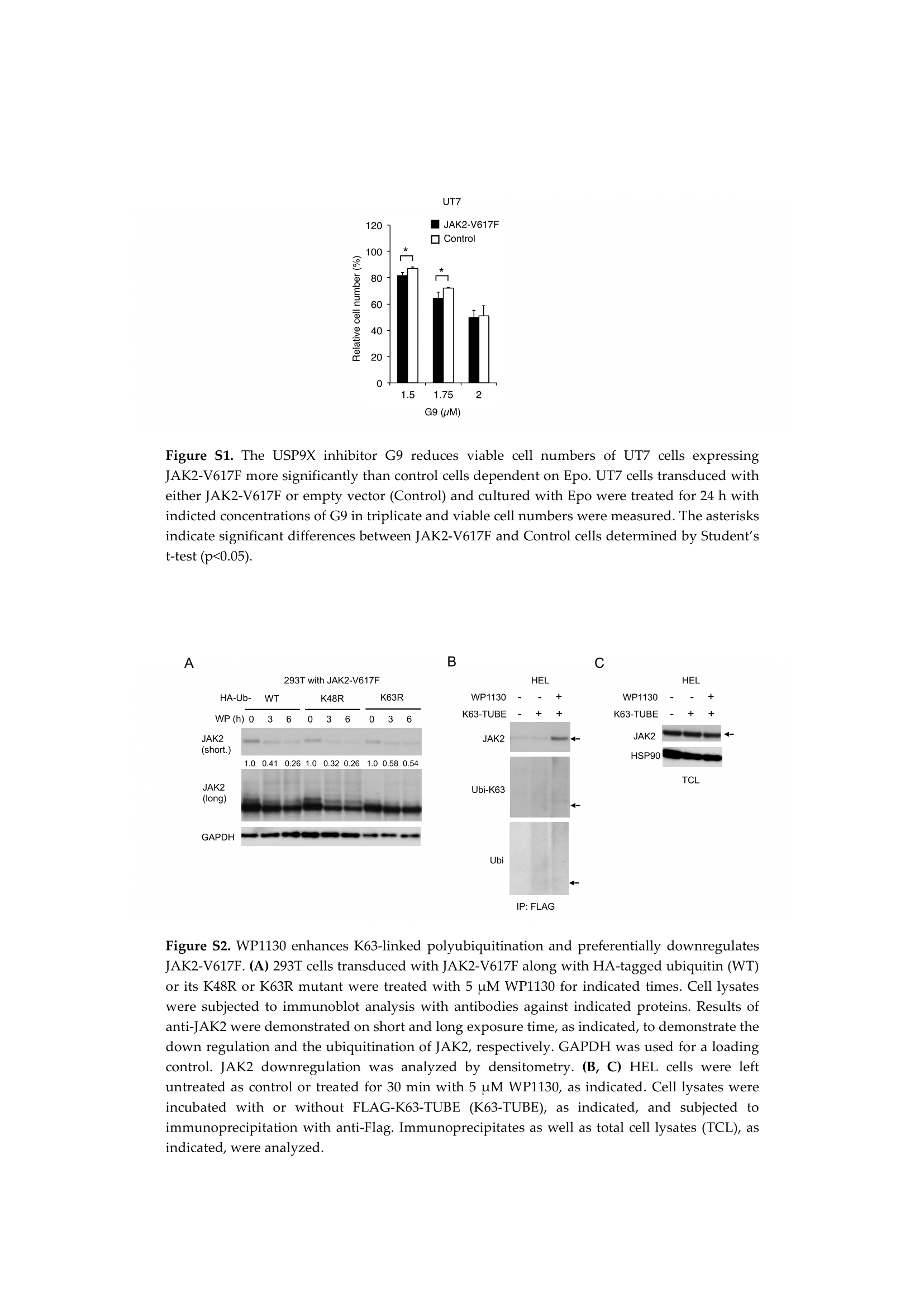


**Figure S1.** The USP9X inhibitor G9 reduces viable cell numbers of UT7 cells expressing. JAK2-V617F more significantly than control cells dependent on Epo. UT7 cells transduced with either JAK2-V617F or empty vector (Control) and cultured with Epo were treated for 24 h with indicted concentrations of G9 in triplicate and viable cell numbers were measured. The asterisks. indicate significant differences between JAK2-V617F and Control cells determined by Student’s t-test (*p* < 0.05).


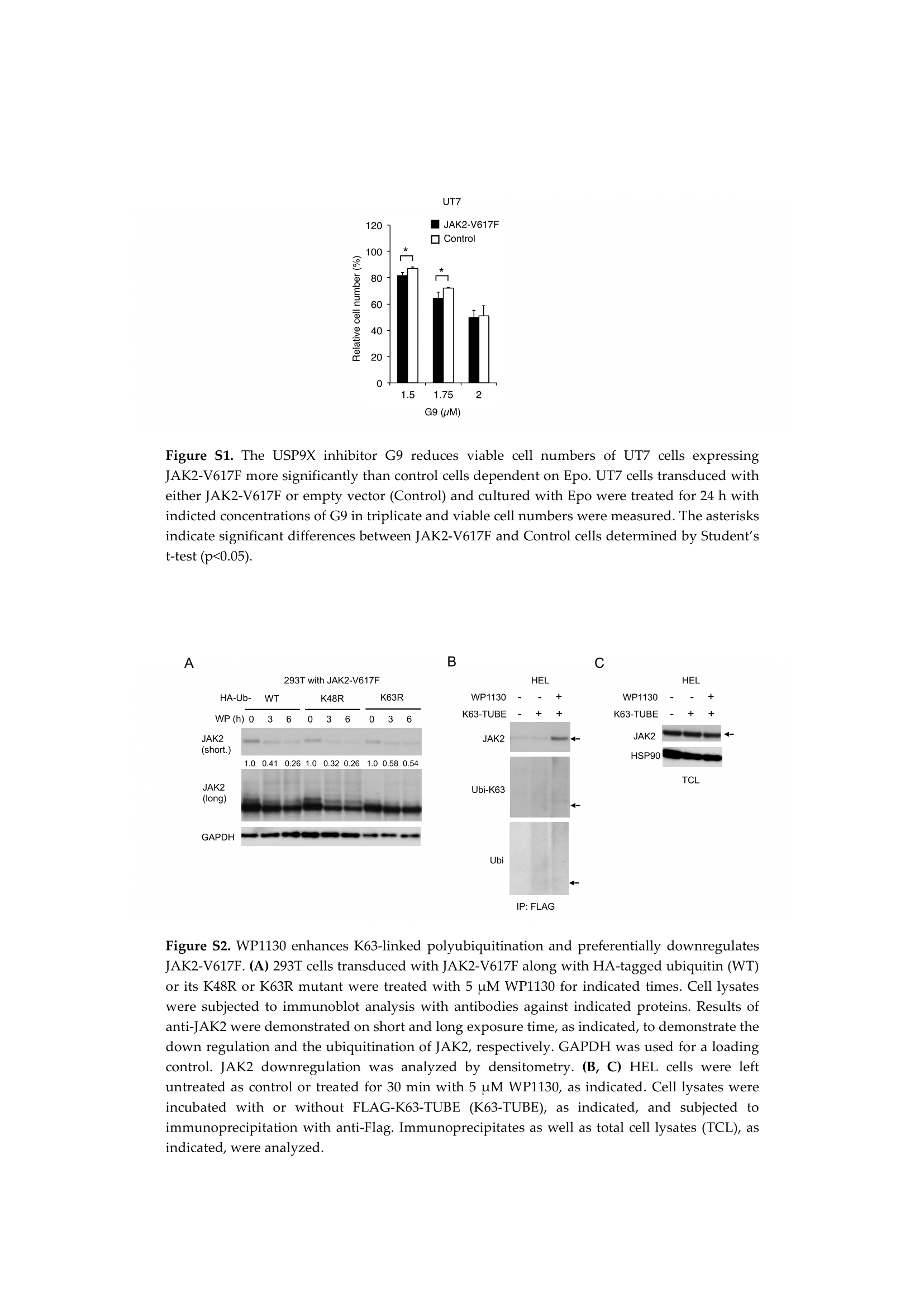


**Figure S2.** WP1130 enhances K63-linked polyubiquitination and preferentially downregulates JAK2-V617F. (**A**) 293T cells transduced with JAK2-V617F along with HA-tagged ubiquitin (WT) or its K48R or K63R mutant were treated with 5 μM WP1130 for indicated times. Cell lysates were subjected to immunoblot analysis with antibodies against indicated proteins. Results of anti-JAK2 were demonstrated on short and long exposure time, as indicated, to demonstrate the down regulation and the ubiquitination of JAK2, respectively. GAPDH was used for a loading control. JAK2 downregulation was analyzed by densitometry. (**B,C**) HEL cells were left untreated as control or treated for 30 min with 5 μM WP1130, as indicated. Cell lysates were incubated with or without FLAG-K63-TUBE (K63-TUBE), as indicated, and subjected to immunoprecipitation with anti-Flag. Immunoprecipitates as well as total cell lysates (TCL), as indicated, were analyzed.


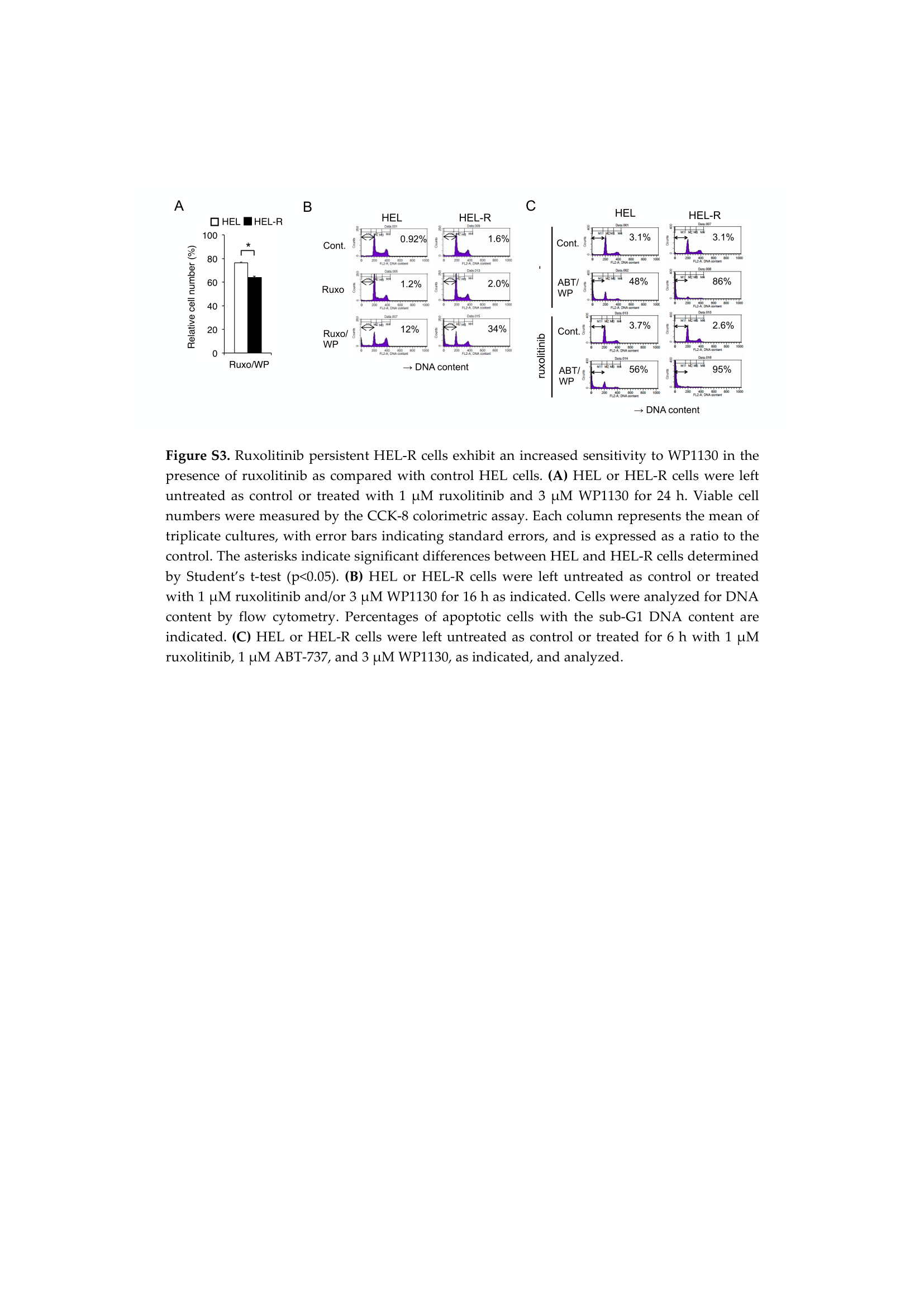


**Figure S3.** Ruxolitinib persistent HEL-R cells exhibit an increased sensitivity to WP1130 in the presence of ruxolitinib as compared with control HEL cells. (**A**) HEL or HEL-R cells were left untreated as control or treated with 1 μM ruxolitinib and 3 μM WP1130 for 24 h. Viable cell numbers were measured by the CCK-8 colorimetric assay. Each column represents the mean of triplicate cultures, with error bars indicating standard errors, and is expressed as a ratio to the control. The asterisks indicate significant differences between HEL and HEL-R cells determined by Student’s t-test (*p* < 0.05). (**B**) HEL or HEL-R cells were left untreated as control or treated with 1 μM ruxolitinib and/or 3 μM WP1130 for 16 h as indicated. Cells were analyzed for DNA content by flow cytometry. Percentages of apoptotic cells with the sub-G1 DNA content are indicated. (**C**) HEL or HEL-R cells were left untreated as control or treated for 6 h with 1 μM ruxolitinib, 1 μM ABT-737, and 3 μM WP1130, as indicated, and analyzed.

| 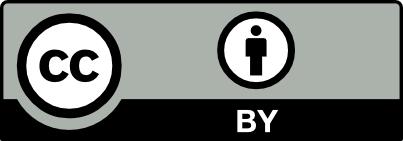 | © 2020 by the authors. Licensee MDPI, Basel, Switzerland. This article is an open access article distributed under the terms and conditions of the Creative Commons Attribution (CC BY) license (http://creativecommons.org/licenses/by/4.0/). |
| --- | --- |
