# Supplementary figures and images for: Inhibition of USP9X Downregulates JAK2-V617F and Induces Apoptosis Synergistically with BH3 Mimetics Preferentially in Ruxolitinib-Persistent JAK2-V617F-Positive Leukemic Cells
Source: Cancers (Basel). 2020 Feb 10;12(2):406. doi: 10.3390/cancers12020406 (PMC7072561; doi:10.3390/cancers12020406)

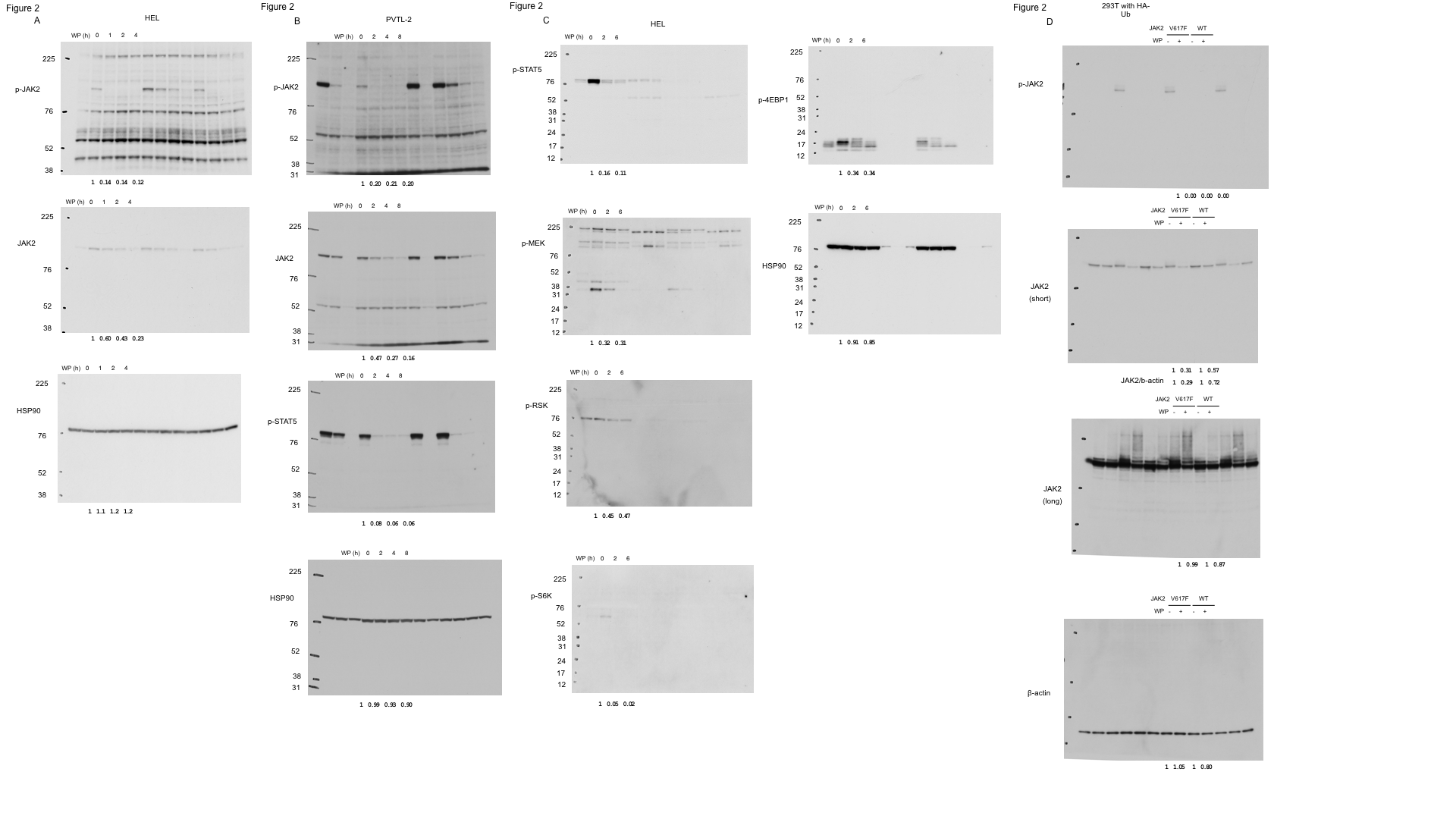

Supplement: Supplementary file 1 [file cancers-12-00406-s001.zip › cancers-702627-suppl-final/original western blot figures/WholeWB.Data.Fig2-1.tiff]

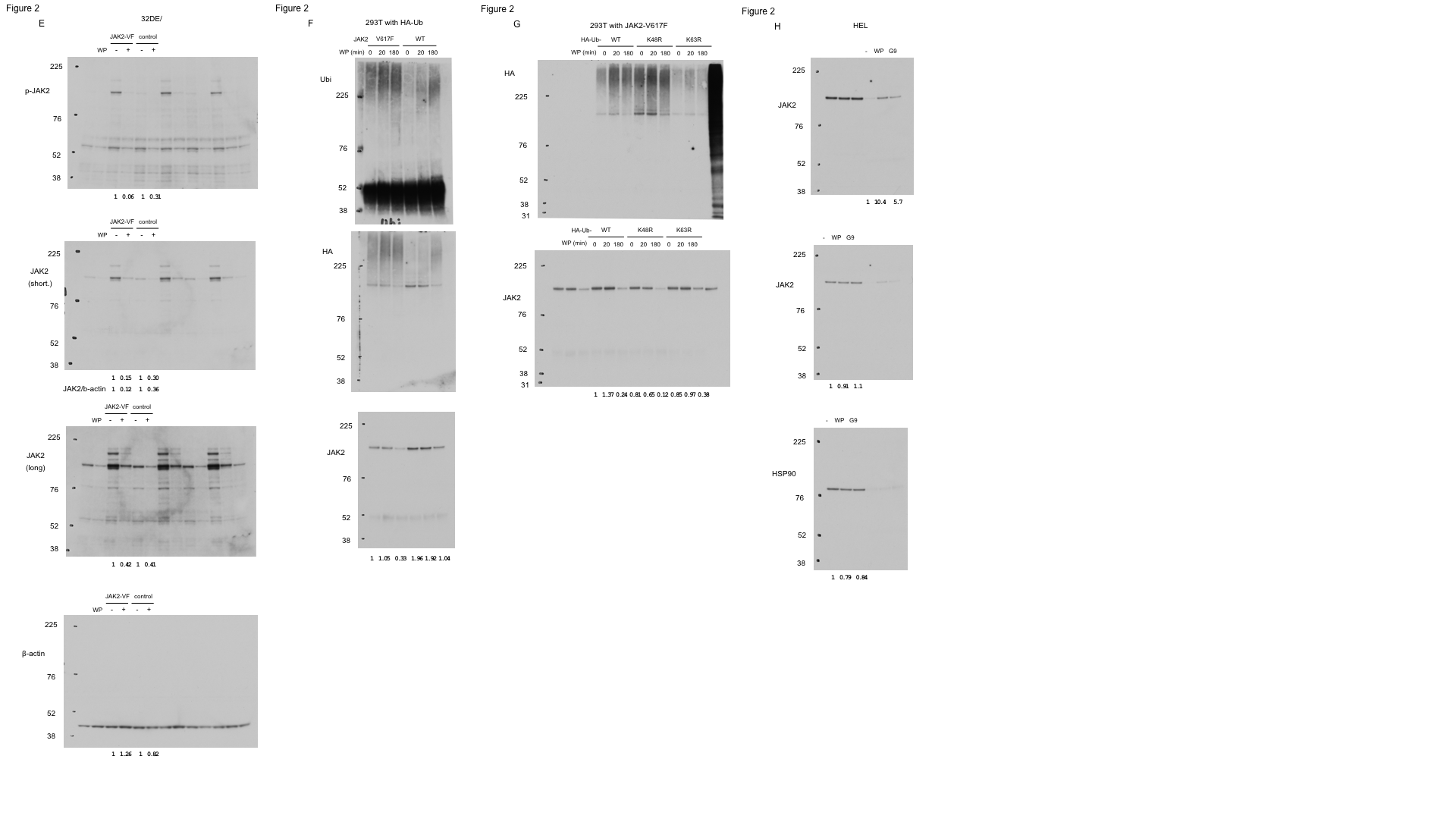

Supplement: Supplementary file 1 [file cancers-12-00406-s001.zip › cancers-702627-suppl-final/original western blot figures/WholeWB.Data.Fig2-2.tiff]

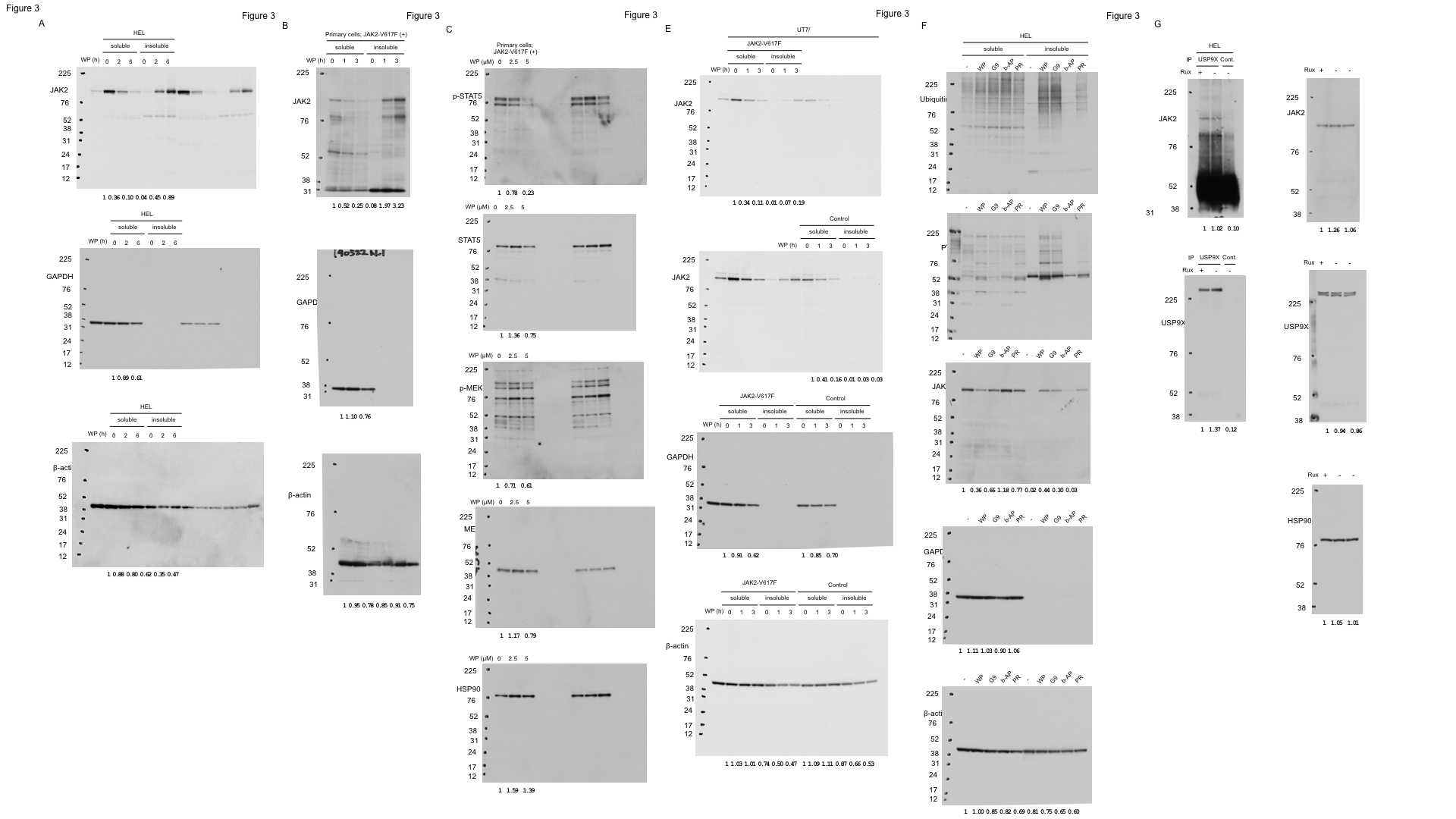

Supplement: Supplementary file 1 [file cancers-12-00406-s001.zip › cancers-702627-suppl-final/original western blot figures/WholeWB.Data.Fig3.tiff]

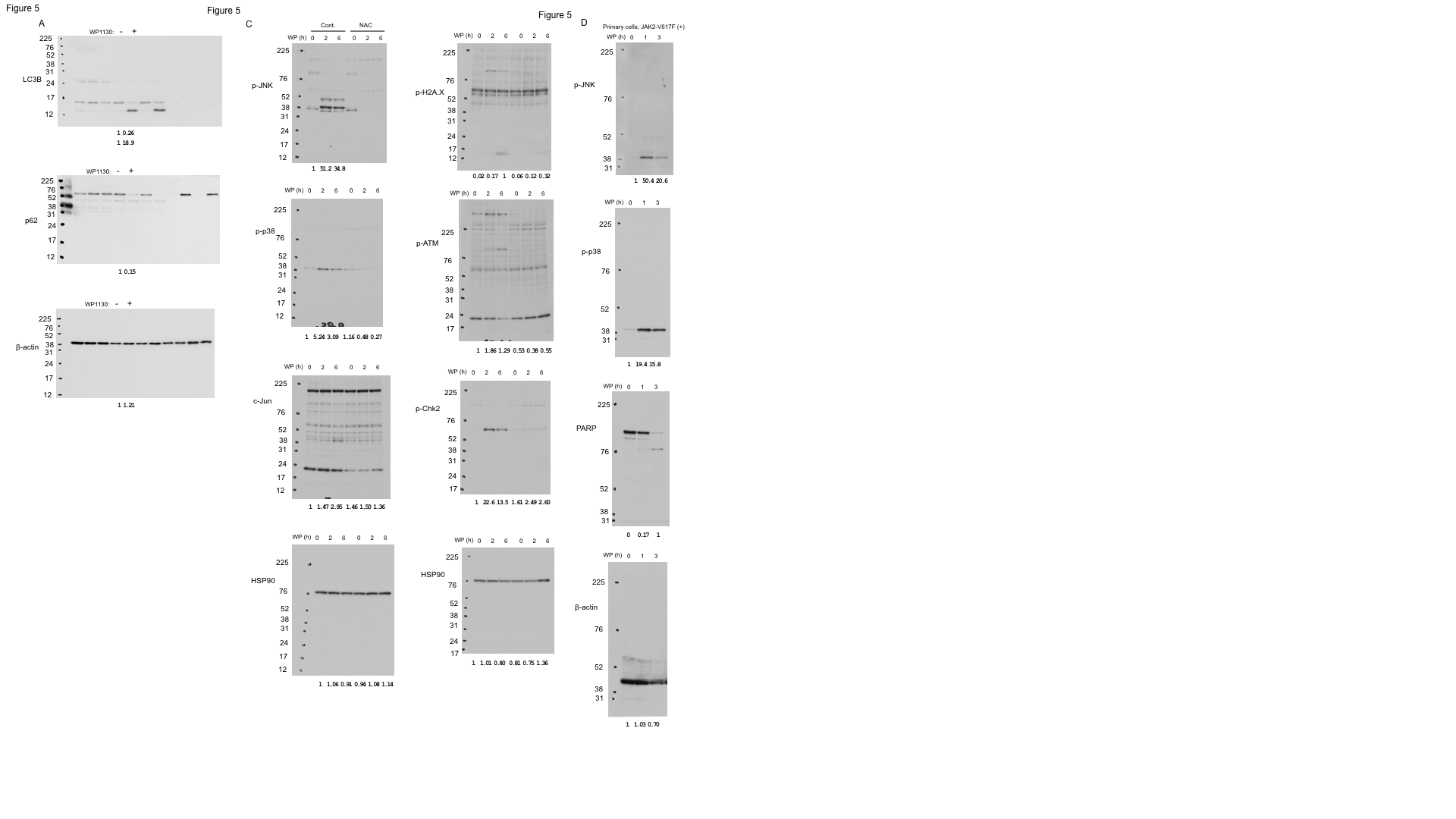

Supplement: Supplementary file 1 [file cancers-12-00406-s001.zip › cancers-702627-suppl-final/original western blot figures/WholeWB.Data.Fig5.tiff]

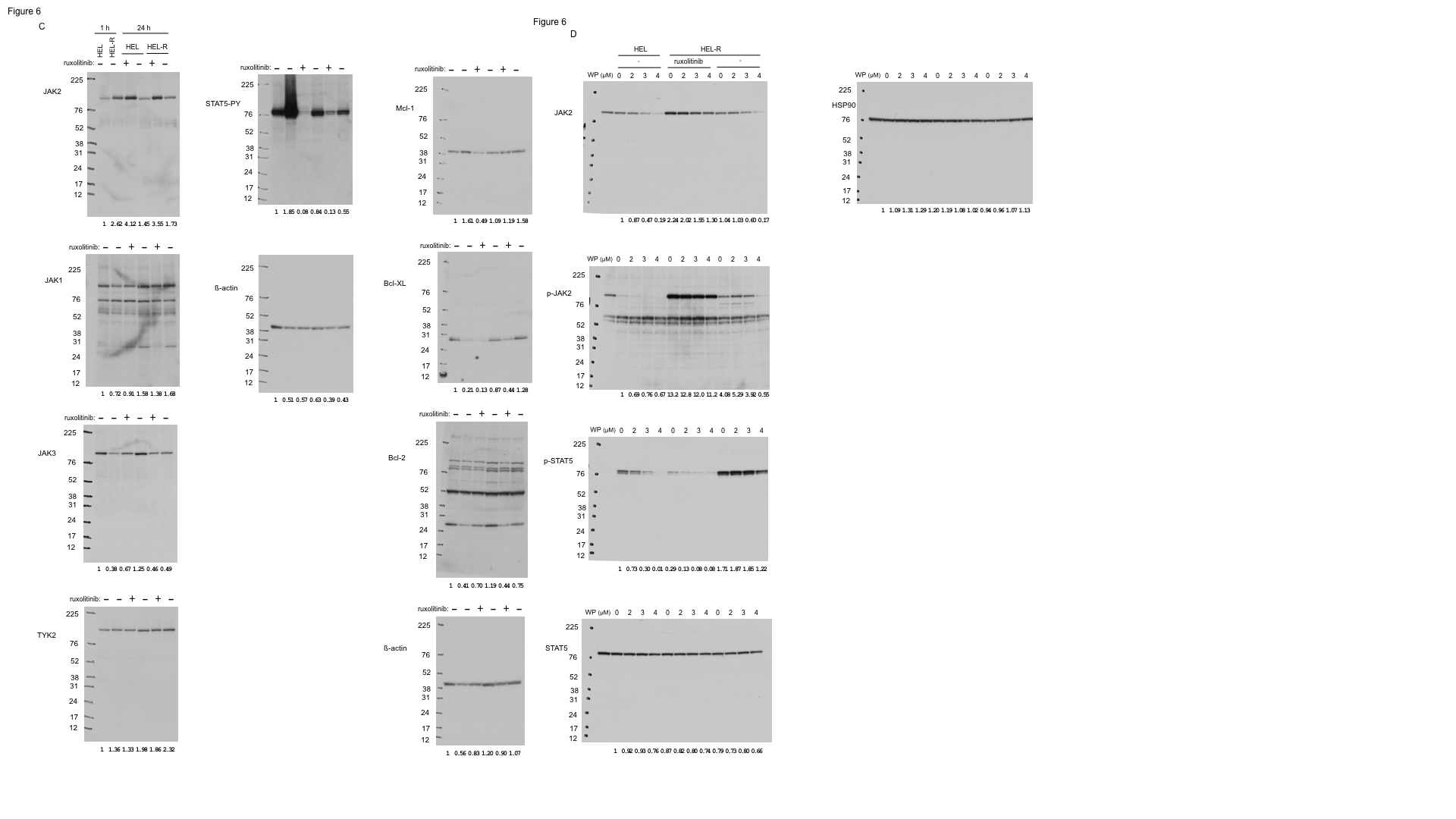

Supplement: Supplementary file 1 [file cancers-12-00406-s001.zip › cancers-702627-suppl-final/original western blot figures/WholeWB.Data.Fig6.tiff]
